# Synthesis of Sustainable Lignin Precursors for Hierarchical Porous Carbons and Their Efficient Performance in Energy Storage Applications

**DOI:** 10.1021/acssuschemeng.3c07202

**Published:** 2024-01-23

**Authors:** Muhammad Muddasar, Misbah Mushtaq, Anne Beaucamp, Tadhg Kennedy, Mario Culebras, Maurice N. Collins

**Affiliations:** † Stokes Laboratories, School of Engineering, Bernal Institute, University of Limerick, Limerick V94 T9PX, Ireland; ‡ Department of Chemical Sciences, University of Limerick, Limerick V94 T9PX, Ireland; § Institute of Material Science, (ICMUV) University of Valencia, Paterna 22085, Spain; ∥ SFI Centre for Advanced Materials and BioEngineering Research, Dublin D02 PN40, Ireland

**Keywords:** lignin, supercapacitors, sodium-ion batteries, batteries, spherical porous carbon, green synthesis, tunable porous carbon, energy storage

## Abstract

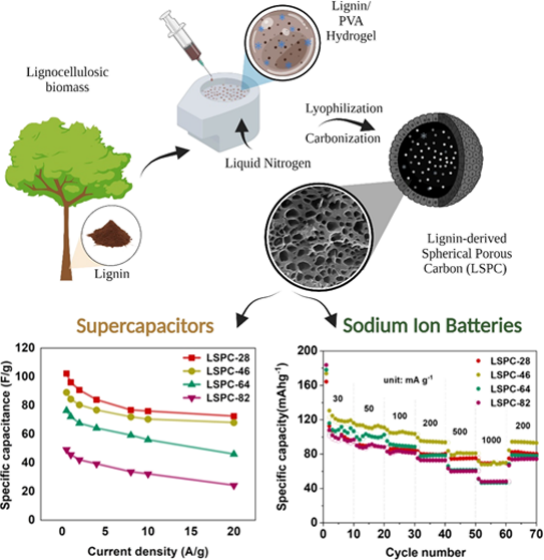

Lignin-derived porous carbons have great potential for energy storage applications. However, their traditional synthesis requires highly corrosive activating agents in order to produce porous structures. In this work, an environmentally friendly and unique method has been developed for preparing lignin-based 3D spherical porous carbons (LSPCs). Dropwise injection of a lignin solution containing PVA sacrificial templates into liquid nitrogen produces tiny spheres that are lyophilized and carbonized to produce LSPCs. Most of the synthesized samples possess excellent specific surface areas (426.6–790.5 m^2^/g) along with hierarchical micro- and mesoporous morphologies. When tested in supercapacitor applications, LSPC-28 demonstrates a superior specific capacitance of 102.3 F/g at 0.5 A/g, excellent rate capability with 70.3% capacitance retention at 20 A/g, and a commendable energy density of 2.1 Wh/kg at 250 W/kg. These materials (LSPC-46) also show promising performance as an anode material in sodium-ion batteries with high reversible capacity (110 mAh g^–1^ at 100 mA g^–1^), high Coulombic efficiency, and excellent cycling stability. This novel and green technique is anticipated to facilitate the scalability of lignin-based porous carbons and open a range of research opportunities for energy storage applications.

## Abstract 2

Sustainable lignin-derived 3D hierarchical porous carbon was synthesized by a novel technique, offering tunable properties and advanced energy storage applications.

## 1 Introduction

Recent decades have seen an increase in the consumption of fossil fuels, causing concerns about the possibility of a global energy crisis. The growth of the renewable energy sector, such as hydropower, wind, and solar energy, has gained considerable attention in recent years as a potential solution to global energy problems.^1−3^ However, the intermittent nature of these energy resources calls for a method of efficient energy storage for later use. Current technological advancements require immediate action for the development of inexpensive, green, sustainable, and high-performing energy storage devices. Supercapacitors (SCs) have gained enormous interest owing to their outstanding attributes of longer life stability (>105 cycles), faster charge–discharge rate (within seconds), and higher power density (10 kW kg^–1^).^4^ Various types of carbonaceous materials, including activated carbons, heteroatom-doped carbons (HDCs), hierarchically porous carbons (HPCs), graphene materials, carbon nanocages, carbide-derived carbons, carbon nanofibers, carbon nanotubes, and carbon dots, have received great interest as SC electrodes.^5^ Sodium-ion batteries (SIBs), another hot research area, have attracted significant research interest because of their abundance and wide distribution as a cost-effective energy storage device for possible large-scale applications.^6^ It has been widely accepted that hard carbon or HPCs can improve the rate of performance of materials in SIBs. However, sustainable carbon materials with hierarchical porous structures for SCs and SIBs remain less developed, and many challenges remain to be addressed.

HPCs are very promising candidates for enhancing the energy storage performance of SCs and SIBs due to their stable 3D framework, interconnected pore structure, and high specific surface area. Coal, petroleum, and their derivatives have been previously utilized for the synthesis of HPCs.^7,8^ The use of these materials, however, has resulted in environmental problems, since they are not sustainable. Biomass-derived HPCs have emerged as prominent energy storage materials because they are environmentally friendly, inexpensive, and sustainable in nature.^9−13^ Lignin is widely considered to be an important natural source for synthesizing carbon materials due to its renewable nature, low cost, and abundance.^14,15^ It is also an attractive carbon precursor material due to its aromatic structure. Additionally, lignin is biodegradable and nontoxic, making it an attractive choice for energy storage applications.^16^ Numerous studies have been conducted recently regarding the potential use of lignin-based carbon materials in energy applications, particularly batteries, fuel cells, and SCs.^17−20^ HPCs can be obtained by direct carbonization of lignin powder but have limited performance in SCs and SIBs. The use of lignin aerogel as a precursor source for the synthesis of 3D HPCs is a unique paradigm in this field.^21^

Lignin aerogel has a three-dimensional pore structure and shows extraordinary qualities including developed porosity, multibranched network structure, and low density.^22^ Several researchers are interested in the development of easy and effective methodologies for the tunable synthesis of lignin-based porous carbon aerogels and converting them into high-performance electrode materials for SCs and SIBs.^23^ The available surface area and pore size of lignin aerogels can be modified by employing some pore-forming agents such as templates (inorganic and polymeric), strong bases (KOH, NaOH), or physical activation methods (CO_2_ activation). Soft templates have attracted extensive attention among all available options because they can directly decompose during the carbonization process, as opposed to using harmful and toxic chemicals for pore formation and etching.^16^ However, soft-template-assisted lignin aerogels are not frequently used as precursors for lignin-derived 3D HPCs, and their potential has not been fully explored. By integrating polymeric templates, a unique pathway for achieving controlled surface area and pore size adjustments in lignin-based materials might be achieved, which has not been extensively investigated previously. Additionally, the conventional methods for synthesizing lignin-derived 3D HPCs encounter challenges, notably the unpredictable porous morphology and the collapse of pores due to the intricate structure of lignin. Therefore, an innovative technique is required that can offer tunable properties to achieve more predictable porous morphologies and tailored properties in lignin-based porous carbon materials.

Herein, for the first time, a green and unique “template-assisted in situ cross-linking” method was successfully established to prepare lignin-based 3D spherical porous carbons (LSPCs). Kraft lignin acts as a carbon source, while PVA acts as a self-cross-linking agent as well as a sacrificial template. Dropwise injection of the lignin/PVA solution in a liquid nitrogen bath yields spherical beads, which undergo lyophilization and carbonization to yield LSPCs. The obtained carbon materials had high specific surface areas with tunable hierarchical morphologies consisting of both micro- and mesopores. LSPCs exhibit excellent SC applications, including superior specific capacitance, excellent rate capability, outstanding cycling stability, and impressive energy density. In addition, when tested as an anode material for SIBs, these LSPCs display good specific capacity (LSPC-46, 110 mAh g^–1^ at 100 mA g^–1^ current density) and are highly stable with excellent Coulombic efficiency. This eco-friendly synthesis technique is expected to streamline the scalability of lignin-based porous carbon, creating numerous research prospects in the field of batteries and SCs.

## 2 Experimental Section

### 2.1 Materials

Kraft lignin (TcB) of *M*_w_ 3153 g/mol was supplied by Tecnaro (GMbH, Ilsfeld, Germany). Sodium hydroxide (NaOH) pellets with ≥98% purity were purchased from AppliChem GmbH (Ilsfeld, Germany). Poly(vinyl alcohol) (PVA) 99% hydrolyzed with an *M*_w_ of 85,000–124 ,000 g/mol was purchased from Sigma-Aldrich (Ireland). Liquid nitrogen was supplied by BOC, Ireland. 1-Ethyl-3-methylimidazolium tetrafluoroborate (EMIMBF_4_) (≥99% purity) was purchased from Sigma- Aldrich (Spain). Sodium trifluoromethanesulfonate (NaCF_3_SO_3_) (purity >98%) and diethylene glycol dimethyl ether (DEGDME, purity >99%) were purchased from Sigma-Aldrich, Ireland.

### 2.2 Preparation of Lignin-Based Spherical Aerogels

PVA powder was first added to the predefined volume of deionized water and stirred thoroughly for 10 min in order to avoid clumps. The solution was then stirred for 30 min at 85 °C to obtain a transparent solution. The obtained PVA solution was cooled down to room temperature and ultrasonicated for 5 min to remove the entrapped air. To facilitate the dissolution of the kraft lignin, the solution was then carefully mixed with 2 M NaOH before adding the prescribed amount of kraft lignin. The homogeneous mixture of lignin/PVA was obtained after 12 h of magnetic stirring at 45 °C. The prepared mixture was injected dropwise into a liquid nitrogen bath using a syringe, and rapid freezing of the solution resulted in tiny spherical beads. Martin Christ α 2–4 LD plus lyophilizer (Focus Scientific, Ireland) was employed at 0.1 mbar pressure, for 24 h, at a condenser temperature of −70 °C for converting the frozen samples into lignin-based spherical aerogels (LSAs).

### 2.3 Synthesis of 3D LSPCs

A tubular furnace (Carbolite Gero 30–3000 °C) was utilized under an inert atmosphere in order to convert the lyophilized LSAs into porous carbon. Samples were initially heated from room temperature to 200 °C at a ramp rate of 5 °C/min. A very slow heating rate of 1 °C/min was adopted between 200 and 300 °C in order to avoid the collapse of pores. Later, the temperature was increased from 300 to 900 °C at a ramp rate of 5 °C/min and maintained at 900 °C for 30 min to complete the carbonization procedure. The carbonized samples underwent a washing process to eliminate any potential impurities, followed by drying at 85 °C for 12 h. Synthesized samples were denoted by LSPC-*XY*, where *X* and *Y* depend on the percentages of PVA and lignin content in the solution. All the formulations of LSPCs are summarized in Table S1.

### 2.4 Material Characterizations

Fourier transform infrared spectroscopy (FTIR) was performed on LSAs using a PerkinElmer (Waltham, MA) Spectrum 100 spectrometer with an attenuated total reflectance (ATR) accessory. A total of 4 scans were conducted per test in a range between 4000 and 650 cm^–1^. Scanning electron microscopy (SEM) was carried out in a Hitachi SU-70 (Hitachi High-Technologies Corporation, Tokyo, Japan) to analyze the morphological characteristics of LSPCs. The accelerating voltage during the SEM observation was 10 kV. Transmission electron microscopy (JEOL JEM-1011 TEM) was used for in-depth morphological analysis of LSPCs. X-ray photoelectron spectroscopy (XPS) technique was used to analyze the surface chemistry of synthesized samples using the Kratos AXIS ULTRA spectrometer. The mono Al Kα 1486.58 eV; 300 W (20 mA, 15 kV) X-ray gun was employed to perform analysis on samples within the 20–30 °C temperature. PANalytical Empyrean instrument equipped with a Cu Kα radiation source (λ = 1.5418 Å) was used for X-ray diffraction (XRD) analysis. The Horiba LabRAM 1A Raman spectrometer equipped with a 514 nm laser was used to measure Raman spectra of porous carbon samples at room temperature in a backscattering configuration. A silicon sample spectrum was used to calibrate all measurements, and the spectrometer was kept in the same position to ensure accuracy. Surface area, pore radius, and volume values were obtained from nitrogen adsorption–desorption isotherms according to the BET theory. The experiments were performed with an ASAP 2010 instrument (Micromeritics Systems) at 77 K. The samples were degassed at 200 °C for 12 h before measurements under nitrogen.

### 2.5 Electrochemical Testing

The electrochemical performance of 3D LSPCs was tested at room temperature using a three-electrode setup. Six M KOH was used as an aqueous electrolyte, a saturated calomel electrode (SCE) was used as the reference electrode, and Pt metal served as the counter electrode. The working electrodes were prepared by mixing LSPCs, carbon black, and poly(vinylidene fluoride) at a mass ratio of 8:1:1 in *N*-methyl-2-pyrrolidone, and then, the mixture was pasted on a nickel foam and dried at 60 °C overnight. The dried-coated nickel foam was pressed at 6 MPa to improve the electrical connections of the active material to the electrode. Cyclic voltammetry (CV) and galvanostatic charge–discharge (GCD) were carried out using an IVIUMnSTAT multichannel electrochemical analyzer. A practical SC device for powering up electrical circuitry (LED in this case) was constructed by sandwiching stainless-steel 316L strips coated with active slurry between glass microfiber filters as separators infiltrated with ionic electrolyte (EMIMBF_4_). The device was further secured by wrapping a parafilm around it to prevent electrolyte leakage and to ensure structural integrity. Mass loading for the supercapacitor was ≈3.2–5.1 mg for each electrode. Formulas used for calculating the performance are written in detail in the Supporting Information.

Furthermore, to evaluate performance of LSPCs as an anode material for SIBs, coin cells were assembled inside an Ar-filled glovebox, and their electrochemical performance was subsequently assessed. The anode was prepared via aqueous slurry processing using a weight ratio of 8:1:1 of active material, carboxymethyl cellulose (CMC), and carbon black, which was coated onto an aluminum foil current collector. Sodium metal was used as the counter electrode and the electrolyte was 1 M sodium trifluoromethanesulfonate (NaCF_3_SO_3_) dissolved in diethylene glycol dimethyl ether (DEGDME). The electrochemical testing of the anode materials was performed using a NEWARE battery tester at different current densities. For sodium-ion battery testing, mass loading was ≈1.1 mg/cm^2^.

## 3 Results and Discussion

**Figure 1:**
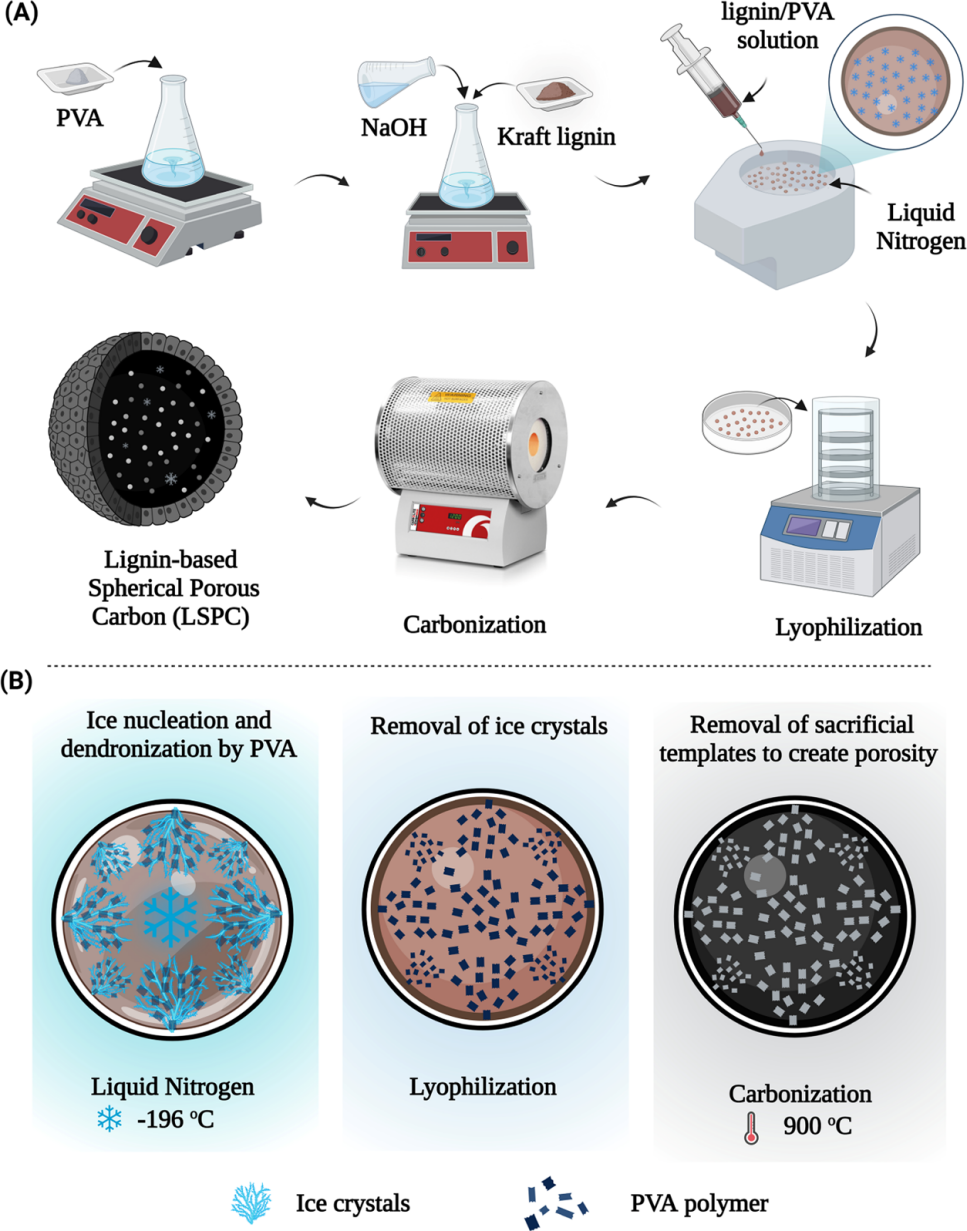
(A) Schematic representation of lignin-based spherical porous carbon (LSPC) synthesis. (B) Schematic illustration of ice crystal growth and dispersion of soft polymeric templates.

3D LSPCs with tunable hierarchically porous structures were synthesized by a “template-assisted in situ cross-linking” strategy. Figure 1A illustrates the green and scalable methodology in a simplified manner. Initially, a PVA solution was prepared, and then, a predefined amount of lignin was added to make a homogeneous solution. Since kraft lignin has a hydrophobic nature, the lignin was unable to mix properly with the PVA solution without the addition of a particular quantity of NaOH solution.^24^ Liquid nitrogen causes the drops of the lignin/PVA mixture to freeze rapidly, resulting in the nucleation of numerous small dendritic ice crystals at the outer surface of the drops. Interestingly, the nucleation of ice crystals occurs in a homogeneous manner because the tiny lignin/PVA droplets are in direct contact with liquid nitrogen, thus offering a considerable driving force for rapid freezing. Dendronization of the ice front is facilitated by the polymeric templates (Figure 1B). The propagation of ice crystals ultimately causes lignin molecules to displace along the direction of polymeric template arrangement, resulting in nanochannel-like morphology from the outer periphery of the droplet toward its center (Figure S2).^25^ It is, however, interesting to note that the amount of PVA strongly influences the degree of polymeric template aggregating near ice crystals, thereby influencing the morphology, porosity, and surface area of the LSPCs. This synthesis technique offers various parameters, including syringe size, injection rate, lyophilization, etc., for optimizing the morphological properties of LSPCs.

**Figure 2:**
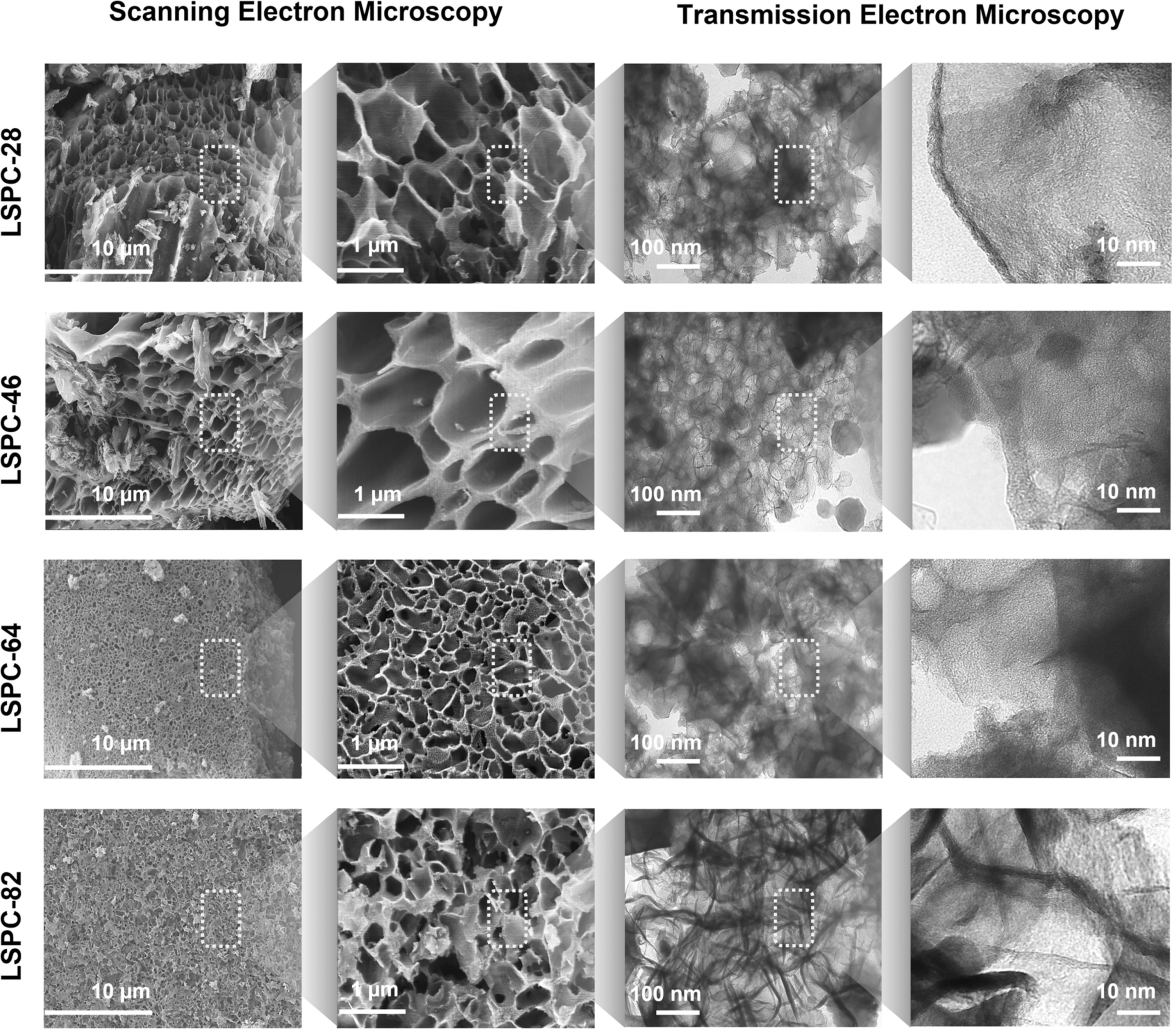
SEM and TEM images of lignin-derived spherical porous carbon samples.

Porous carbon samples were obtained after the carbonization of LSAs. SEM and TEM techniques were employed for analyzing the morphological differences between synthesized LPSCs with different lignin concentrations. It is obvious from SEM images that all of the synthesized samples have a porous morphology (Figure 2). Despite this, the results indicate that with the change in polymeric template concentration, the pore morphology of LSPC samples changes significantly. A hierarchically aligned porous channel-like morphology was observed in LSPC samples with less PVA concentration (LSPC-28 and LSPC-46). On the other hand, samples with high concentrations of polymeric templates have randomly distributed pores of various sizes over the surface. This difference is associated with the viscosity of the lignin/PVA solution, which affects the uniform propagation of the ice front. The solution with a low PVA content has a lower viscosity and offers negligible dendronization of the ice front, resulting in the linear propagation of the ice crystals and alignment of templates. Increasing PVA concentration in solution leads to higher viscosity that impedes the linear propagation of the ice front, resulting in dendronization of ice crystals in all directions and random distribution of polymeric templates. Hence, the observed trend in SEM images is consistent with the mechanism illustrated in Figure 1B. Additionally, lignin samples containing high levels of polymeric content, especially LSA-82, collapse during carbonization, creating cracks and irregularities within the carbon structure, reducing the uniformity of porous structure, as shown in Figure 2 (LSPC-82). PVA templates were removed after carbonization, and the orientation of the templates determines the morphological pattern of the pores in LSPCs. In addition, TEM images also confirmed the presence of hierarchical morphologies and indicated that all LSPCs possessed numerous nanopores of varying sizes. This “template-assisted in situ cross-linking” method represents a pivotal approach for fabricating lignin-based 3D spherical porous carbons, encompassing macropores, mesopores, and micropores. The intricate interplay of these diverse pore sizes is essential for electrolyte infiltration, ion diffusion, and adsorption, tailoring their performance for advanced energy storage applications. An important thing to note here is that the morphology and pore size of LSPCs can be easily controlled by adjusting the PVA concentration.

**Figure 3:**
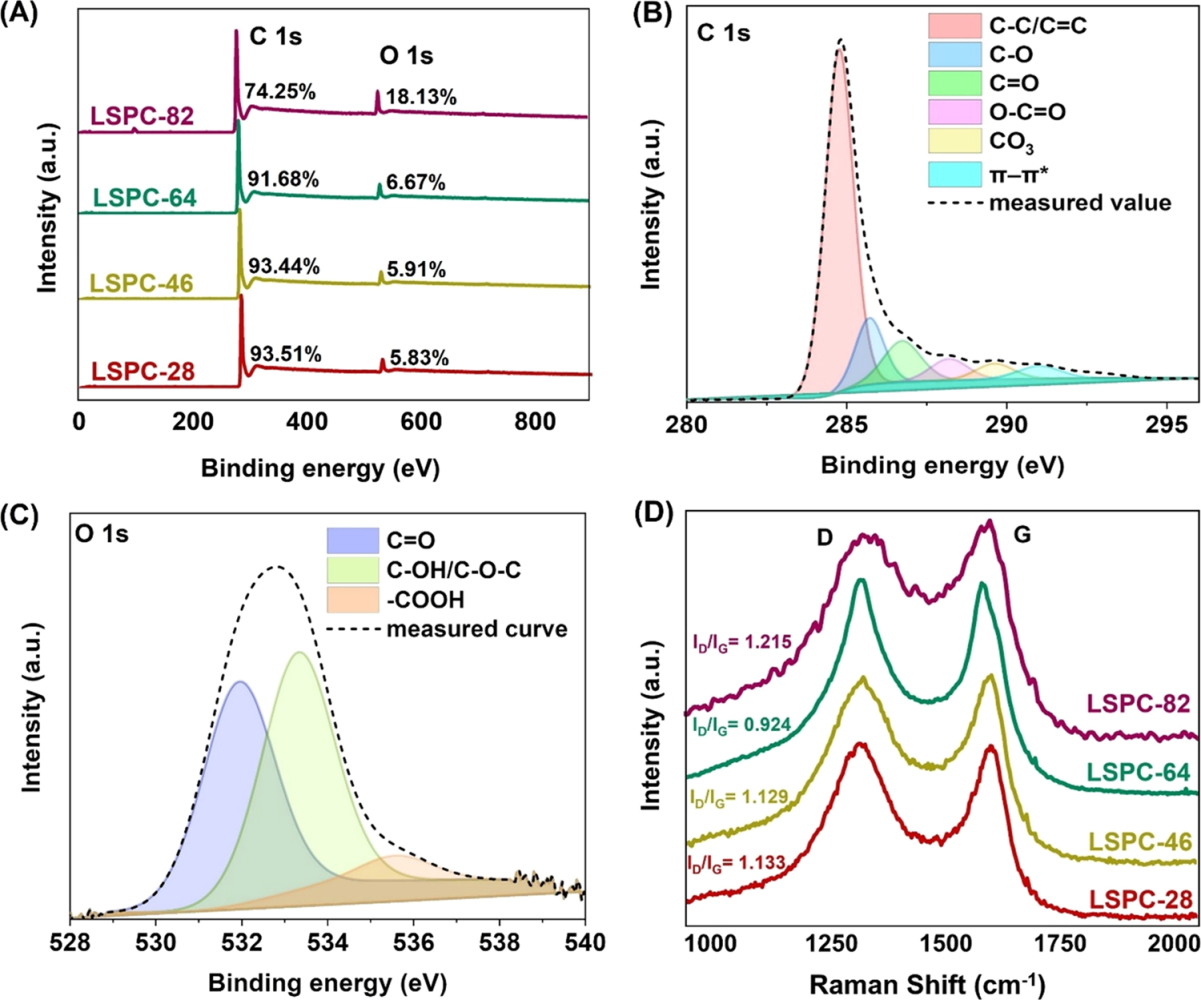
XPS analysis of LSPC samples: (A) survey spectrum of all samples with atomic composition of C and O, (B) detailed C 1s core spectra of LSPC-28, (C) O 1s core spectra of LSPC-28, and (D) Raman spectra of all LSPC samples.

The surface chemistry of the 3D LSPCs was characterized by XPS. The full spectrum acquired shows that all of the samples except LSPC-82 were mostly composed of carbon and oxygen with slightly varying concentrations, Figure 3A. However, the excess concentration of PVA in LSPC-82 contributes to the higher atomic concentration of oxygen (18.13%). This higher oxygen content can lead to decreased electrical conductivity, which can negatively affect the energy storage performance of the LSPC-82 material.^26^ The high-resolution XPS spectrum of LSPCs was also examined in order to determine the detailed chemical states of individual elements, and the results are illustrated in Figures 3B,C, S3, and S4. The C 1s spectra of LSPC-28 sample are fitted by six individual peaks located at around 283.8, 284.8, 285.7, 286.7, 288.2, 289.6, and 291.0 eV corresponding to the C–C/C=C, C–O, C=O, O–C=O, CO_3_, and π–π*(satellite peak), respectively (Figure 3B). The high-resolution spectra of the O 1s of LSPC-28 are also further resolved into three different peaks (Figure 3C), revealing the existence of oxygen-containing functional groups including C=O (quinone-type groups at 532.0 eV), C–OH/C–O–C (phenol/ether groups at 533.3 eV), and –COOH (chemisorbed oxygen or carboxylic groups at 535.6 eV). It is important to note that the presence of C=O functional groups can enhance the hydrophilicity of the electrode materials and the groups are electrochemically active to introduce extra pseudocapacitance.^27,28^

The effect of variations in the concentration of PVA and lignin on the structure, crystallinity, and degree of graphitization of 3D LSPCs was assessed using Raman spectroscopy and XRD. Raman spectra were performed on the LSPCs for graphitic structure assessment, and the results are presented in Figure 3D. In each sample analyzed, two distinct peaks were observed, which were attributed to the characteristic D and G bands present at 1344 and 1595 cm^–1^, respectively. The band positions and intensities can provide information about the degree of graphitization and structural ordering in the sample. The D band at 1344 cm^–1^ is correlated with sp^2^ carbon with hydrogen sites and oxygen-containing groups along with sp^3^ defects in hexagonal graphitic layers. Meanwhile, the G band signifies the in-plane motion of ordered sp^2^ graphitic carbon.^29^ The Raman spectra of carbon materials are generally used to analyze the structural defects using the *I*_D_/*I*_G_ intensity ratio. The resulting value provides information about the level of defects in the carbon material, as the presence of defects leads to an increase in the *I*_D_/*I*_G_ ratio.^30^ The ratio was calculated by carefully fitting the raw data from the D and G bands (Figure S5). As a result, values of 1.133, 1.129, 0.924, and 1.215 were calculated for LSPC-28, LSPC-46, LSPC-64, and LSPC-82, respectively. It is evident from the results that defects reach the maximum value in the sample with the highest PVA concentration. This is attributed to irregular arrangements of polymeric templates, as discussed in the previous paragraph.

**Figure 4:**
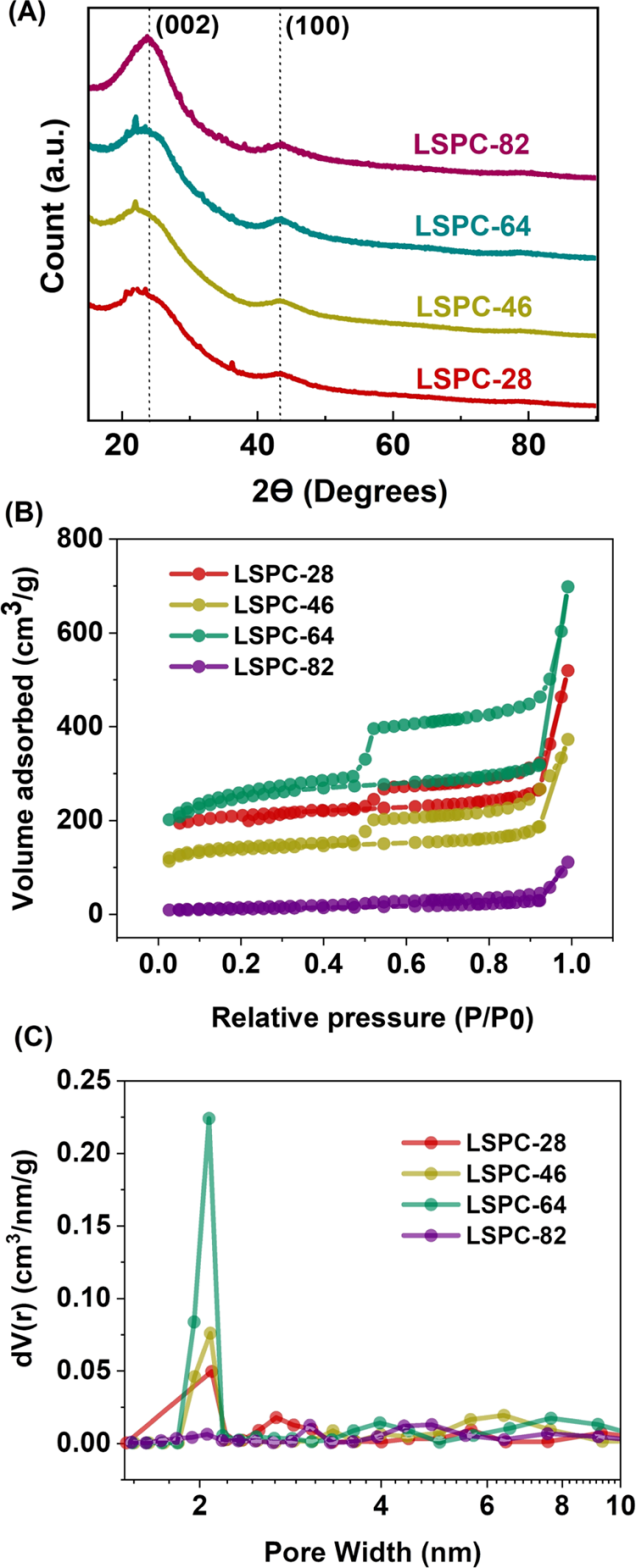
(A) XRD spectra, (B) N_2_ adsorption–desorption isotherms, and (C) pore size distribution curves of all lignin-derived porous carbon samples.

The wide-angle XRD spectrum of the synthesized LSPCs is depicted in Figure 4A. A similar diffraction pattern can be observed in all LSPC samples, showing a significant peak at 22° and a slight peak at 43°, corresponding to the (002) and (100) crystal planes of graphitelike carbon and cubic amorphous carbons, respectively.^31^ The intensities of these peaks can provide information about the relative abundance of these structures and their degree of ordering. The presence of these broad peaks indicates the existence of amorphous carbon structure with abundant defects.^32^ Bragg’s law was used to calculate the interlayer spacing for (002) graphitelike carbon planes. The value of interlayer spacing decreases as the concentration of PVA increases, ranging from 2.0578 to 1.824 Å for LSPC-28 and LSPC-82, respectively. These values are in good agreement with the HRTEM results of LSPC-28 (Figure S9).

The pore structure properties of the LSPC samples were studied by N_2_ adsorption–desorption measurements and are presented in Figure 4B and Table S2. All LSPCs except for LSPC-82 display typical type IV isotherms with simultaneous presence of H1 and H4 type hysteresis loops within the relative pressure range of 0.45–1.^33^ These results indicate the presence of numerous mesopores with cylindrical and narrow silt-shaped morphologies, which are believed to result from the arrangement of polymeric templates during rapid freezing, resulting in a hierarchical porous morphology as evidenced by SEM analysis, especially in LSPC-28 and LSPC-46. Likely, the high concentration of polymeric templates in the LSPC-82 sample collapsed during carbonization, resulting in the lack of hysteresis and fewer mesopores. The specific surface areas of LSPC-28, LSPC-46, LSPC-64, and LSPC-82 were determined to be 646.2, 426.4, 790.5, and 35.6 m^2^/g, respectively. This surface area is associated with the fact that LSPC-28 and LSPC-46 have a channel-like morphology arising from an ice crystal arrangement. The high surface area of LSPC-28 (646.2 m^2^/g) is due to the formation of tiny ice crystals and PVA chain alignment, forming narrow channels after carbonization, contributing to the higher surface area. LSPC-46 has a slightly lower surface area as increased PVA aligns more polymer chains, resulting in PVA agglomeration and the creation of larger channels. However, in LSPC-64, higher PVA content disrupts ice crystal uniformity, resulting in a random distribution of PVA templates due to phase separation, yielding a nonuniform porous morphology and the highest surface area (790.5 m^2^/g). All of these observations are in good agreement with SEM analysis of samples. It is important to mention that even though LSPC-64 has the highest surface area, nonuniformity in pores might influence the electrochemical performance. In contrast, the last sample, LSPC-82, contains a significant amount of PVA, causing it to collapse during carbonization and yielding the lowest surface area.

The pore size distribution (PSD) was also characterized using N_2_ adsorption–desorption isotherms, and the results are shown in Figure 4C. It is important to note that all samples, except LSPC-82, show a significant PSD peak between 1.5 and 2.5 nm, indicating the presence of micropores. The observed microporosity can be attributed to the activation of carbon due to the presence of NaOH, which serves as a lignin-dissolving agent in the initial mixture. This decomposition releases hydroxyl ions that interact with surface carbon atoms, resulting in the formation of micropores as shown in TEM images.^34^ Furthermore, PSD peaks ranging between 2 and 10 nm also confirm the presence of mesopores of varying sizes. The BET results demonstrate that the LSPC samples have a unique 3D hierarchical structure due to the presence of interconnected micro- and mesopores distributed throughout the samples. Micropores serve as sites for the accumulation of charge, whereas mesopores serve as channels for shortening the distance between ions during diffusion, resulting in increased SC efficiency.^35^

**Figure 5:**
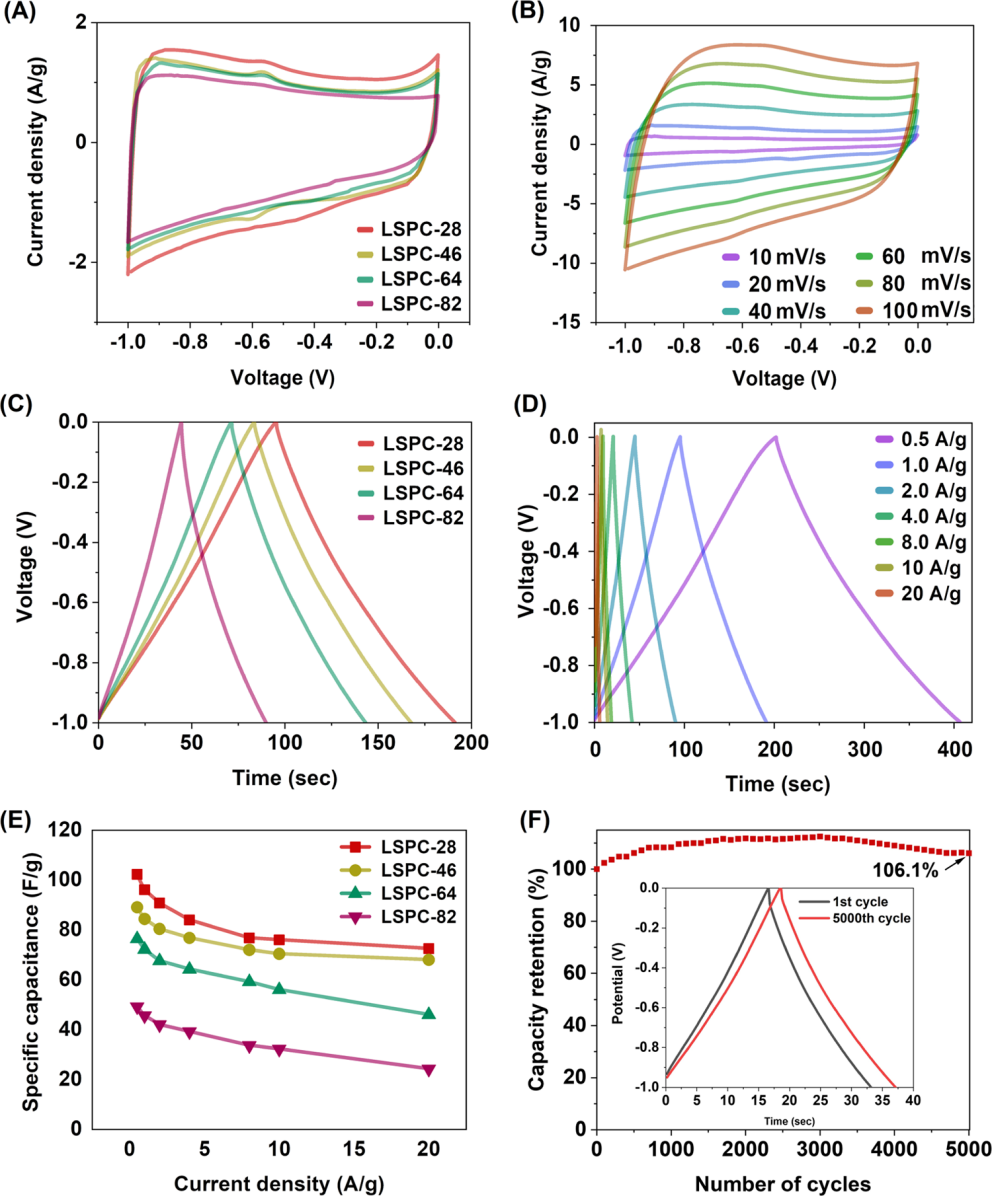
Electrochemical testing of LSPC samples in 3-electrode configuration. (A) CV comparison at 20 mV/s scan rate, (B) CV curves of LSPC-28 at different scan rates, (C) GCD comparison at 1 A/g current density, (D) GCD curves of LSPC-28 at different current densities, (E) specific capacitance of all LSPC samples at different current densities, and (F) cyclic performance of LSPC-28 at 5 A/g.

The electrochemical analysis was conducted using 6.0 M KOH electrolyte on LSPCs in a 3-electrode system arrangement. The CV analysis of all LSPCs was performed in the −1 to 0 V voltage window with the scan rate set to 20 mV/s (Figure 5A). A quasi-rectangular shape was exhibited during the CV curves, indicating that the charge storage occurs due to electric double-layer capacitive (EDLC) behavior.^5^ Additionally, some samples exhibit tiny faradaic humps associated with surface reactions between electrolyte ions and oxygen-containing groups that cause pseudocapacitive behavior.^21,36^ The largest integral area of the CV curve for LSPC-28 samples suggests that it has the highest specific capacitance compared to others. This performance is particularly notable since LSPC-28, while possessing a moderate specific surface area, achieves its superior electrochemical performance from its unique macroscopic morphology.

In samples LSPC-28 and LSPC-46, there is a notable phenomenon where the ice crystals grow uniformly, resulting in channel-like macroscopic pores, as shown in Figure 2. These channel-like macroscopic pores exhibit a high degree of interconnectivity with the smaller mesopores and micropores present in the material. It is important to note that this interconnected pore structure facilitates the efficient infiltration of electrolytes as well as provides a substantial amount of surface area for the adsorption of ions.^37,38^ Moreover, with this 3D interconnected channel-like morphology, the LSPC-28 provides stacking porosities so that ions can diffuse rapidly and provides conductive networks to shorten electron transport paths, which are essential to the enhancement of supercapacitor performance. On the other hand, for sample LSPC-64, despite having a substantial overall surface area and relatively fewer defects in its smaller nanoporous morphology, there is a distinct issue with the macroscopic pores. These larger pores are randomly distributed throughout the material and often exhibit closed or noninterconnected characteristics, as observed through SEM morphology (Figure 2). This limited interconnectivity of the macroscopic pores in sample LSPC-64 restricts the efficient infiltration of electrolyte into the material, hindering the ability of ions to be adsorbed effectively. As a result, the supercapacitor’s performance is adversely affected, as it cannot utilize the available surface area for ion adsorption optimally. Additionally, the oxygen content in the samples also increases with the increase in PVA content in samples. Therefore, the samples with low oxygen content result in improved electrical conductivity and overall electrochemical performance.^39^

The detailed CV analysis of the LSPC-28 sample was carried out at various scan rates, and the results are displayed in Figure 5B. A rectangular-like CV curve was generated for all scans with slight variations, indicating that EDLC is the primary factor that determines the total capacitance. Additionally, pseudocapacitive behavior also contributes to increased capacitance, as observable faradaic humps are seen in CV curves. A higher scan rate of 100 mV/s also results in an almost rectangular CV curve, indicating easy ion diffusion and quick charge transportation ability within the LSPC-28 electrodes.^40^ GCD technique was also used to evaluate the electrochemical performance of LSPCs. It is evident from Figure 5C that all samples exhibit nearly symmetrical triangular GCD curves at 1 A/g current density, which corresponds to EDLC behavior.^41^ In comparison to other samples, LSPC-28 has a longer discharge time, indicating the highest specific capacitance, which agrees with the CV results. In addition, LSPC-28 samples were tested for their ability to store charge at lower and higher current densities (0.5–20 A/g). It is also evident from the nearly isosceles triangular GCD curves at all current densities that EDLC behavior accounts for most of the capacitances involved (Figure 5D).^42^ There is, however, a slight bending in the discharge curve at low current densities, attributed to the surface faradic reactions occurring at the electrode–electrolyte interface.^43^ Nyquist plot of LSPCs also reveals low charge-transfer resistance for all LSPCs (LSPC-28 = 1.22 Ω, LSPC-46 = 1.43 Ω, LSPC-64 = 1.94 Ω, and LSPC-82 = 2.1 Ω) and equivalent series resistance (∼2.64 Ω, 2.89 Ω, 3.02 Ω, 3.25 Ω for LSPC-28, 46, 64, and 82 respectively). Further, the slope of the line (in the low-frequency region) increased in inclination to the imaginary axis, prominently for LSPC-28, indicating rapid charge transfer, excellent capacitance behavior, and low diffusion resistance (Figure S6). The discharge time of GCD curves at several current densities was used to evaluate the specific capacitances of all LSPC samples, and the results are plotted in Figure 5E. In comparison with LSPC-46 (89.1 F/g), LSPC-64 (76.4 F/g), and LSPC-82 (49.5 F/g), LSPC-28 shows a superior specific capacitance of 102.3 F/g at 0.5 A/g. This superior specific capacitance of LSPC-28 should be ascribed to the large specific surface area and 3D interconnected micro- and mesoporous structures. Moreover, LSPC-28 remains stable at 20 A/g and maintains 70.8% of its initial capacitance (72.5 F/g), suggesting an improved rate of ion diffusion.^44^ LSPC-28 has also been evaluated in terms of its cycling performance using the GCD at a rate of 5 A/g, as demonstrated in Figure 5F. Interestingly, a specific capacitance retention of 106.1% at the end of 5000 cycles was recorded. This impressive stability can be attributed to the hierarchical porous morphology of the electrode material. Initially, only the larger pores and mesopores were accessible to the electrolyte, limiting the utilization of micropores. However, over time, K^+^ ions gradually infiltrated the micropores, contributing to the development of substantial electric double layers.^45^ As a result, LSPC-28 exhibited a consistent capacitance, showcasing excellent reversibility and cycle stability throughout the repeated charge–discharge process.^33^ Additionally, LSPC-28 maintains its triangular shape after 5000 cycles, further demonstrating its superior durability.

**Figure 6:**
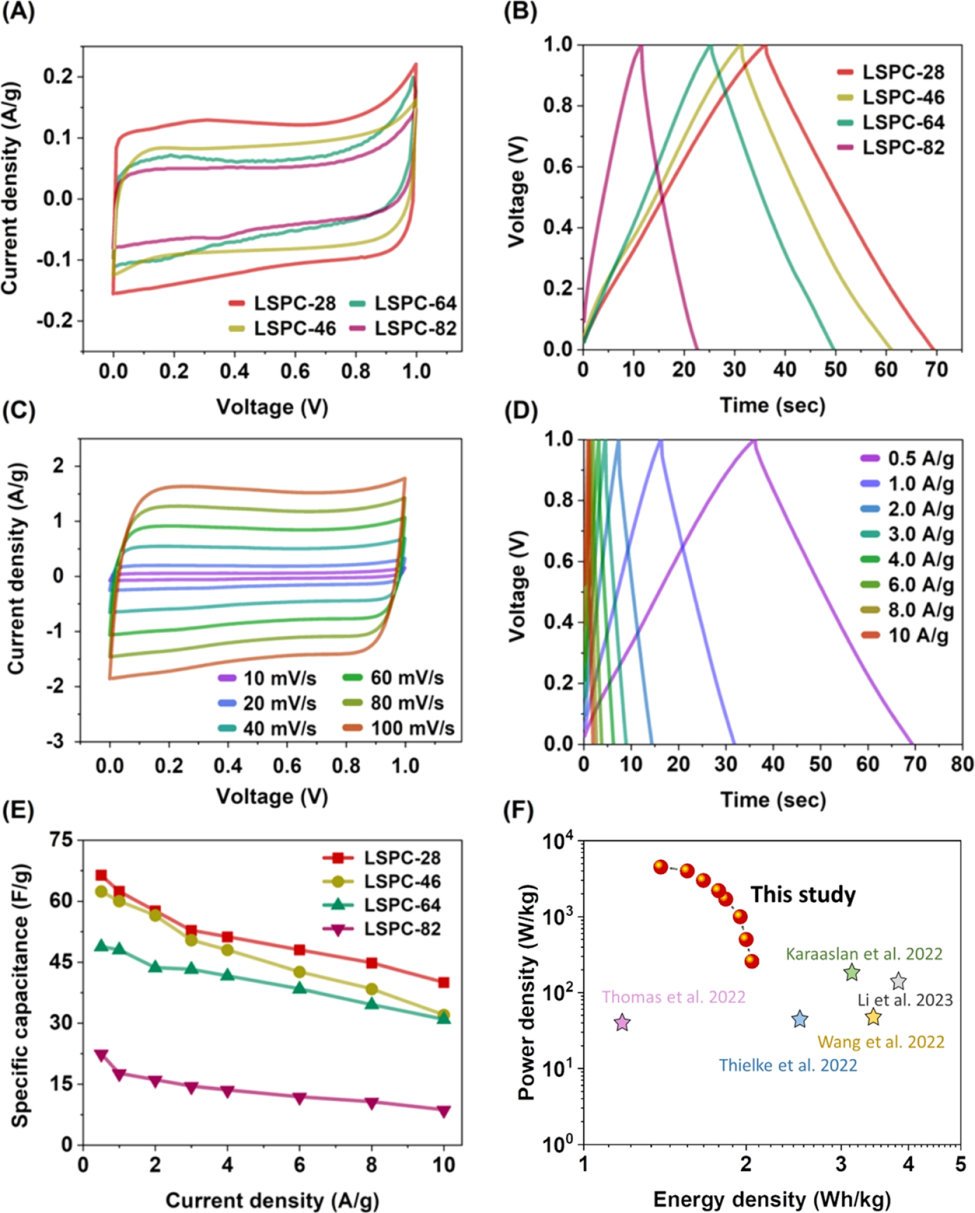
Electrochemical testing of LSPC samples: symmetric SCs configuration in 6 M KOH electrolyte. (A) CV comparison at 10 mV/s scan rate, (B) GCD comparison at 0.5 A/g current density, (C) CV curves of LSPC-28 at different scan rates, (D) GCD curves of LSPC-28 at different current densities, (E) specific capacitance of all LSPC samples at different current densities, and (F) Ragone plot for LSPC-28 compared with recent studies.^48−52^

The performance of the LSPC electrodes was also evaluated in a symmetrical two-electrode setup in a 6 M KOH electrolyte (Figure 6). A quasi-rectangular CV curve is observed for all samples at 10 mV/s scan rate, as shown in Figure 6A. Additionally, symmetrical triangle-like shapes were seen on the GCD curves at 0.5 A/g for all samples, verifying the dominance of EDLC charge storage behavior (Figure 6B). A comparison with other samples shows that LSPC-28 shows the highest current response in CV and the longest discharge time in GCD, which indicates the highest specific capacitance. The CV curves of the LSCP-28 electrodes are shown in Figure 6C in a range of scan rates starting from 100 to 10 mV/s. The well-maintained quasi-rectangle shapes with slight variations confirm the superior stability of EDLC SCs. The LSPC-28 electrodes exhibit symmetrical triangular-shaped GCD curves for varying current densities, which is another desirable feature of EDLC SCs (Figure 6D).^46^

Figure 6E shows the specific capacitance of all LSPC samples calculated at different current densities (0.5–10 A/g). The LSPC-28 exhibits the highest specific capacitances at all current densities, measuring 66.4 F/g at 0.5 A/g. Moreover, LSPC-28 remains stable at 10 A/g and maintains 60.3% of its initial capacitance (40.1 F/g). The cross-linked porous structure of LSPC-28 serves as a rapid pathway for ion transfer, which results in high-rate capability.^43,47^ The Ragone plot shows that the symmetric SC fabricated by LSPC-28 electrodes delivers an excellent energy density of 2.1 Wh/kg at 250 W/kg. Moreover, even at a peak power density of 4500 W/kg, the 1.4 Wh/kg energy density is still maintained (Figure 6F). In summary, the exceptional electrochemical performance of LSPC-28 can be attributed to its high specific surface area, 3D hierarchical porous structure, and uniform distribution of micro- and mesopores. These structural features enable the material to exhibit superior cycling stability, high specific capacitance, and good energy density, all of which are essential for high-performance SC applications.

**Figure 7:**
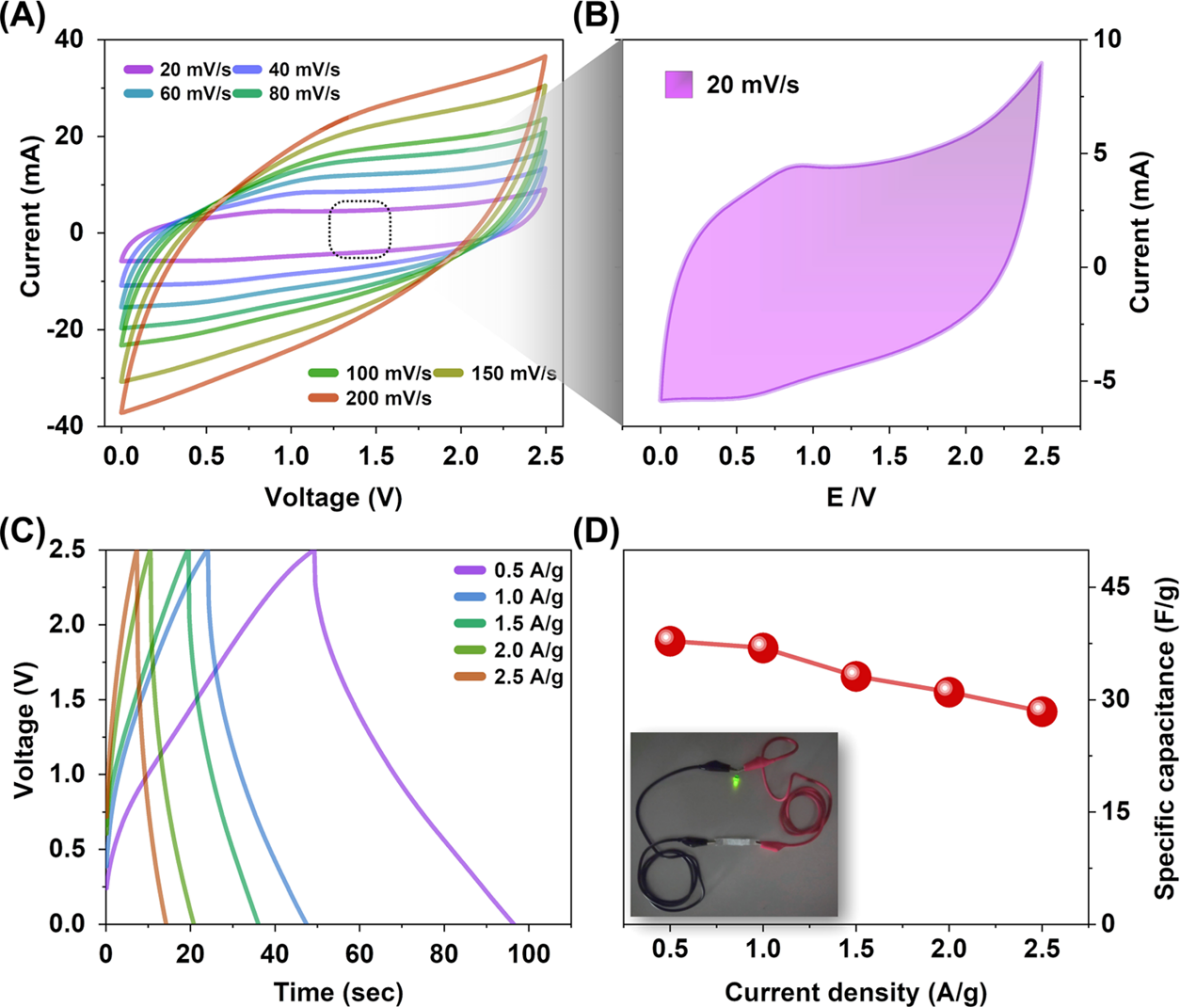
Electrochemical testing of LSPC samples: symmetric SCs configuration in the EMIMBF4 electrolyte. (A) CV curves of LSPC-28 at different scan rates, (B) CV curve of LSPC-28 at 20 mV/s scan rate, (C) GCD curves of LSPC-28 at different current densities, and (D) specific capacitance of the sample at different current densities (inset: 2.0 V green LED powered by symmetrical SCs).

The practical applications of SCs in aqueous electrolytes are limited due to their low operating voltage range of 1–1.8 V.^43^ To overcome this limitation and create a viable SC for powering electrical circuitry, this study developed a symmetrical SC using LSPC-28 material and a neat EMIMBF_4_ ionic liquid as the electrolyte. Unlike aqueous electrolytes, EMIMBF_4_ offers a wider operating voltage range of 2.5 V, which allows for an increased voltage window and improved performance.^53^ The cyclic voltammetry (CV) curves obtained from the assembled device exhibited a quasi-rectangular shape, indicating a significant contribution of capacitance through the EDLC phenomenon (Figure 7A,B). The CV curves also exhibited remarkable stability, maintaining their characteristic shape even at an elevated scan rate of 200 mV/s. Furthermore, the galvanostatic charge–discharge (GCD) profiles of the SC displayed nearly isosceles triangle shapes, even at a current density of 2.5 A/g (Figure 7C). These characteristics are associated with the hierarchical porous structure of LSPC-28, enabling efficient ion diffusion and rapid charge transportation. The specific capacitance of the fabricated device was determined to be 37.9 F/g at a current density of 0.5 A/g. Although this value is lower than that achieved with a symmetrical SC filled with 6 M KOH electrolyte (66.4 F/g at 0.5 A/g), it is still sufficient to light a green LED (1.8 V) and demonstrates its potential as an effective SC material (Figure 7D). The lower specific capacitance of the device can be attributed to the significantly higher ionic electrolyte resistance of EMIMBF_4_ compared to aqueous electrolytes, primarily due to the slower flow of ions caused by the electrolyte’s high viscosities, which limit the charge transportation speed as well as the accessibility of the electrolyte to smaller pores in the electrode surface.^54^

**Figure 8:**
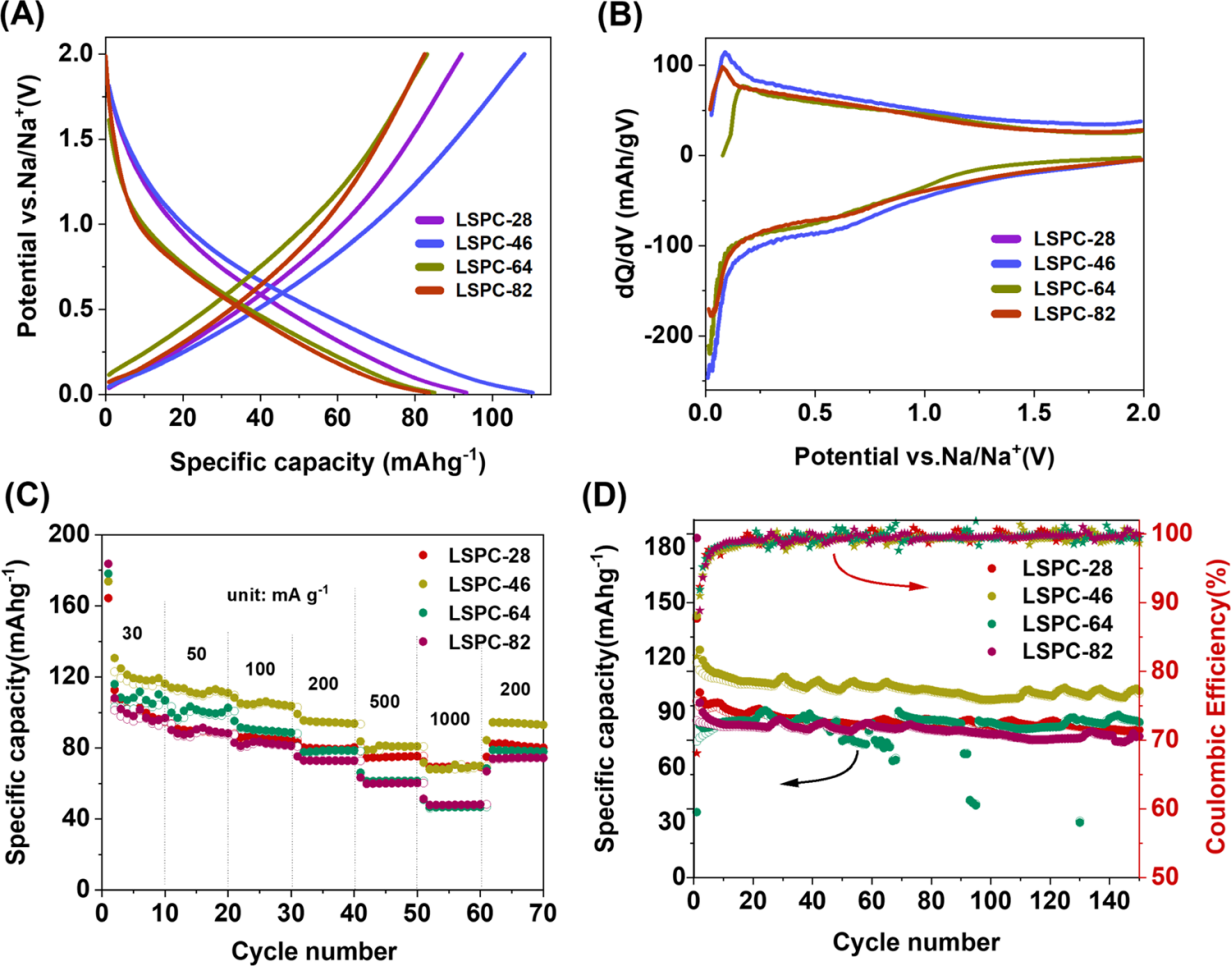
Electrochemical testing of LSPC samples as SIB anode materials. (A) GCD curves of 10th cycle of LSPC samples, (B) differential capacity plot of LSPC electrodes (electrodes were cycled at 100 mA g^–1^ in the potential range of 0.01–2.0 V), (C) rate capabilities of LSPC samples at different current densities, and (D) cyclic performance of LSPC samples at 100 mA g^–1^.

Furthermore, LSPCs have also been studied as anode materials for SIBs in order to pave the way toward a sustainable future of energy. The galvanostatic charge/discharge voltage profiles of the LSPCs at the current density of 100 mA g^–1^ in a potential window of 0.01–2.0 V (vs Na/Na^+^) are displayed in Figure 8A. There is a subtle slope near 1.0 V and a plateau region near 0.0 V indicative of the sodiation and desodiation of ions, respectively. Figure 8B displays the dQ/dV curves which show corresponding broad peaks between 1.0 and 0.2 V related to the gradual voltage drop and a sharper peak <0.2 V related to the plateau region. The reduction peak corresponds to the insertion of sodium ions into the LSPC electrode during the sodiation process and the oxidation peak is due to their extraction during the desodiation process. The exact sodium storage mechanism is unclear; however, the voltage profiles can be divided into two portions, i.e., 2.0–0.2 and 0.2–0.01 V. The sloped region between 2.0 and 0.2 V is ascribed to the insertion of Na ions into the edges or pores of the LSPCs, while the sloped region in the range of 0.2–0.01 V is attributed to the intercalation of Na ions into the graphitic layers domains with a large *d* spacing. For the LSPCs investigated in this study, the voltage profile does not show a long plateau near 0.0 V versus Na/Na^+^ in contrast to previous studies on hard carbon anodes.^55−57^

The rate capability of each sample was performed at 30, 50, 100, 200, 500, and 1000 mA g^–1^ with the results presented in Figure 8C. LSPC-46 was the outstanding performer, retaining 130, 114, 108, 103, 99.14, and 83.7 mAh g^–1^, respectively, at each current density. When the current density reverted to 200 mA g^–1^ after 60 cycles, the capacity returned to 110 mAh g^–1^. Whereas, at the same current densities, LSPC-28, LSPC-64, and LSPC-82 deliver lower charge capacities as compared to LSPC-46. Long-term cycling was performed at 100 mA g^–1^ as shown in Figure 8D. LSPC-46 electrode exhibits a stable capacity around 110 mAh g^–1^ with nearly 98.86% Coulombic efficiency (C.E.) over 150 cycles. LSPC-28, LSPC-64, and LSPC-82 exhibit capacities of 80, 84, and 77 after 100 cycles and C.E. values of 99.24, 99.24, and 99.15%, respectively. A selection of voltage profiles for each type of LSPC over this extended cycling period is presented in Figure S7. The second, 10th, 50th, 100th, and 150th charge/discharge curves are displayed and almost overlap, indicating that the sodiation and desodiation processes of LSPCs possess good reversibility during cycling. Compared to previous published works, Tables S3 and S4 show the performance of these LSPCs in supercapacitors and sodium-ion batteries. Overall, the excellent capacity retention, good rate capability performance, and long-term cycling stability of the LSPCs suggest that the material is a promising candidate as a SIB anode.

## 4 Conclusions

The present study utilizes a green and unique “template-assisted in situ cross-linking” method to prepare lignin-based 3D LSPCs. The as-prepared LSPCs possess a unique 3D hierarchical porous morphology due to the rapid freezing process and the use of soft templates (PVA). Most of the synthesized samples possess excellent specific surface areas (426.6–790.5 m^2^/g) along with hierarchical micro- and mesoporous morphologies. When tested in SC applications, the LSPC-28 was found to have a superior specific capacitance of 102.3 F/g at 0.5 A/g and excellent rate capability with 70.3% capacitance retention at 20 A/g. Additionally, it exhibits an impressive cycling stability of 106.1% after 5000 cycles and a commendable energy density of 2.1 Wh/kg at 250 W/kg due to its 3D hierarchical porous morphology. Furthermore, these materials (LSPC-46) demonstrate promising performance as an anode material of SIBs with high reversible capacity (110 mAh g^–1^ at 100 mA g^–1^), high Coulombic efficiency, and excellent cycling stability. This eco-friendly synthesis technique is expected to streamline the scalability of lignin-based porous carbon, creating numerous research prospects in the field of batteries and SCs.

## Supporting Information Available

Detailed methodology; FTIR analysis of LSAs; SEM analysis of LSAs; XPS spectras; Raman spectra and fitting; Nyquist plot of LSPC-28 in 2-electrode configuration; GCD of LSPCs in sodium-ion batteries; carbon yield of LSPC-28; HRTEM image of LSPC-28; digital images of LSPC-28 as-prepared, freeze-dried, and carbonized; BET analysis of LSPC samples; and comparison of this study with previous studies (PDF)
