# Supplementary material for: Synthesis of Sustainable Lignin Precursors for Hierarchical Porous Carbons and Their Efficient Performance in Energy Storage Applications
Source: ACS Sustain Chem Eng. 2024 Jan 23;12(6):2352–63. doi: 10.1021/acssuschemeng.3c07202 (PMC10865442; doi:10.1021/acssuschemeng.3c07202)
Supplement: Supplementary file 1 — sc3c07202_si_001.pdf [file sc3c07202_si_001.pdf]

Supporting information

Synthesis of sustainable lignin precursors for hierarchical porous carbons and their efficient performance in energy storage applications

Muhammad Muddasar [a], Misbah Mushtaq [b], Anne Beaucamp [a], Tadhg Kennedy [b], Mario Culebras [c], and Maurice N. Collins [ad*]*

[a] Stokes Laboratories, School of Engineering, Bernal Institute, University of Limerick, Limerick, V94 T9PX, Ireland.

[b] Department of Chemical Sciences, University of Limerick, Limerick V94 T9PX, Ireland

[c] Institute of Material Science, (ICMUV) University of Valencia, Paterna, , 22085, Spain.

[d] SFI Centre for Advanced Materials and BioEngineering Research, Dublin, D02 PN40, Ireland.

[*] Email : mario.culebras@uv.es, maurice.collins@ul.ie

Number of Pages: 16

Number of figures: 10

Number of tables: 4

Figure S1**:** FTIR spectra of precursor materials and lignin-derived spherical aerogels
Figure S2: Morphological analysis of lignin-derived spherical aerogels (cross-section) using SEM.
Figure S3: XPS analysis: Detailed C 1s core spectra of LSPCs
Figure S4: XPS analysis: Detailed O 1s core spectra of LSPCs
Figure S5: Raman Spectrum of all LSPCs with D and G band fitting
Figure S6: Nyquist plot of LSPCs in electrode configuration 6M KOH electrolyte (10 mHz – 100 KHz)(amplitude 10 mV)
Figure S7: Galvanostatic charge/ discharge curves of LSPCs samples (A) LSPC-28, (B) LSPC-46, (C) LSPC-64, (D) LSPC-82

Figure S8: TGA and DTG curve for LSA-28
Figure S9: HRTEM image of LSPC-28
Figure S10: Digital images of LSPC-28 as prepared, freeze-dried, and carbonized.

Table S1: Summary of precursors for lignin-derived 3D SPCs

Sample	PVA (g)	Lignin (g)	DI water (mL)	2 M NaOH (mL)
**LSPC-28**	0.3	1.2	7.5	7.5
**LSPC-46**	0.6	0.9	7.5	7.5
**LSPC-64**	0.9	0.6	7.5	7.5
**LSPC-82**	1.2	0.3	7.5	7.5

Electrochemical performance of LSPCs in supercapacitors:

For ink preparation, this study employed mortar and pestle grinding as a method for controlling the particle sizes of the carbon samples. The formula for calculating the specific capacitance of 3D LSPCs from GCD graphs in 3-electrode configuration is given below in eq (1):

$$C_s = \frac{I \times \Delta t}{m \times \Delta V} \quad \rightarrow \quad (1)$$

where C_s (F/g), I (A), Δt (s), ΔV (V), and m (g) stand for the specific capacitance, current density, discharge time, potential window, and mass of active material, respectively.

In a two-electrode system, CV and GCD curves were measured using the asymmetrical supercapacitors in 6.0 M KOH as the electrode. The specific capacitance for the single electrode (C_{sp}), energy density (E; Wh/kg), and power density (P; W/kg) were obtained as follows:

$$C_{sp} = \frac{4I \times \Delta t}{M \times \Delta V} \quad \rightarrow \quad (2)$$

$$E = \frac{C_{sp} \times \Delta V^2}{8 \times 3.6} \quad \rightarrow \quad (3)$$

$$P = \frac{3600 \times E}{\Delta t} \quad \rightarrow \quad (4)$$

Where ΔV (V) refers to the voltage range, M (g) represents the total mass of the active materials on two working electrodes, Δt (s) is the discharge time, and I (A) is the discharge current. The

electrochemical test was conducted in duplicate, and there was only a ±5% uncertainty in the results.

FTIR ANALYSIS

FTIR spectra of the pristine lignin, PVA, and freeze-dried LSAs in the range from 4000 cm^{-1} to 750 cm^{-1} are shown in Figure S1. In the spectrum of pristine kraft lignin, aromatic skeletal vibrations create resonance bands, principally associated with the C-H stretching at 2850-32000 cm^{-1}, C=C stretching around 1620 cm^{-1}, and in-plane bending of -CH around 1410 cm^{-1} [1]. Pristine lignin also contains phenolic and hydroxyl groups characterized by a broad peak of -OH stretching and a peak of -CO stretching around 3000 and 1250 cm^{-1}, respectively [2]. A high-intensity peak of -OH stretching around 3285 cm^{-1} along with transmittance peaks of C-H stretching, bending, and -CO stretching is evident in the FTIR spectra of PVA, which is indicative of intermolecularly bonded hydroxyl functional groups within the aliphatic structure [3]. The FTIR spectra of all LSAs have similar characteristics to those of their precursors. There is, however, an interesting shift in the peak of -OH stretching in response to lignin and PVA concentrations from 3378 cm^{-1} to 3282 cm^{-1}. PVA and lignin form new hydrogen bonds when the concentrations of the precursors change, resulting in this band shift [4].

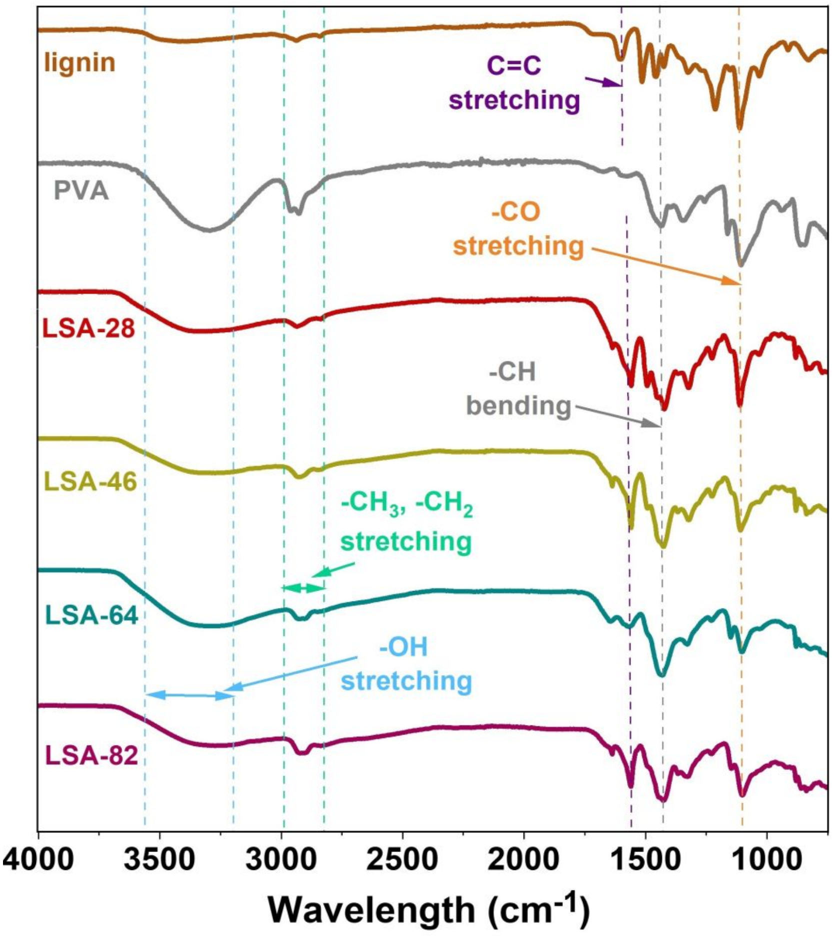

Figure S1: FTIR spectra of precursor materials and lignin-derived spherical aerogels

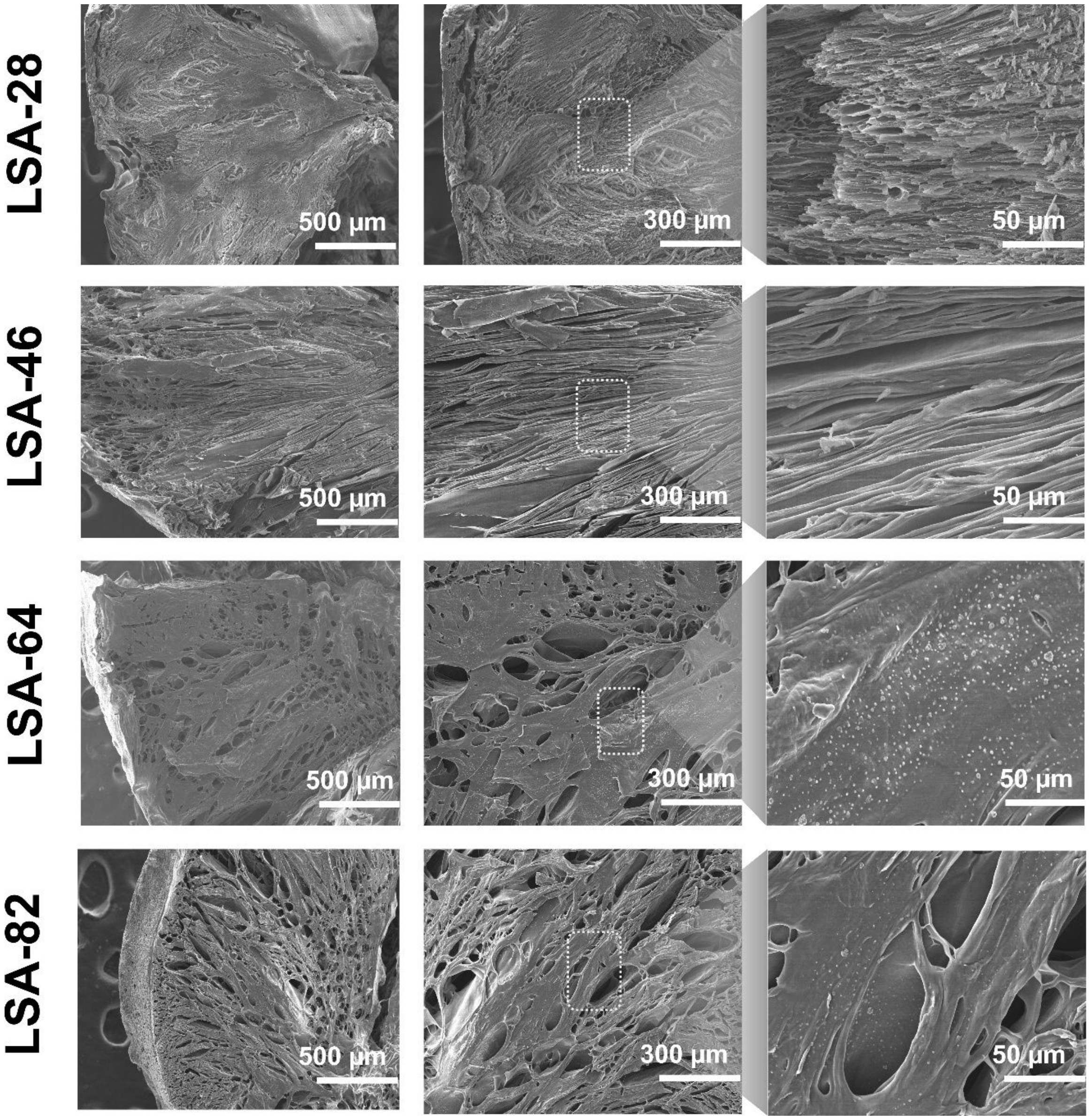

Figure S2: Morphological analysis of lignin-derived spherical aerogels (cross-section) using SEM.

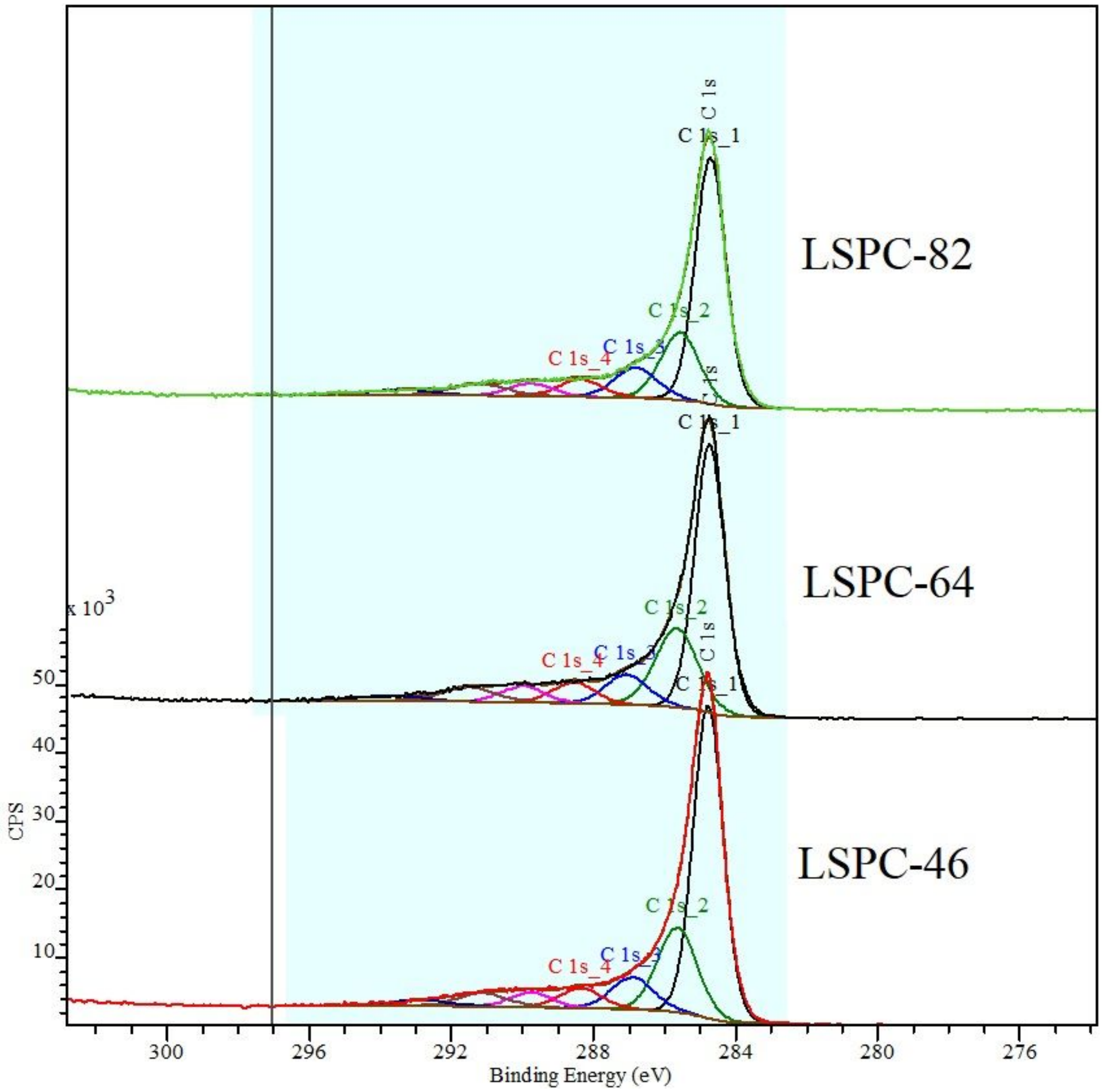

Figure S3: XPS analysis: Detailed C 1s core spectra of LSPCs

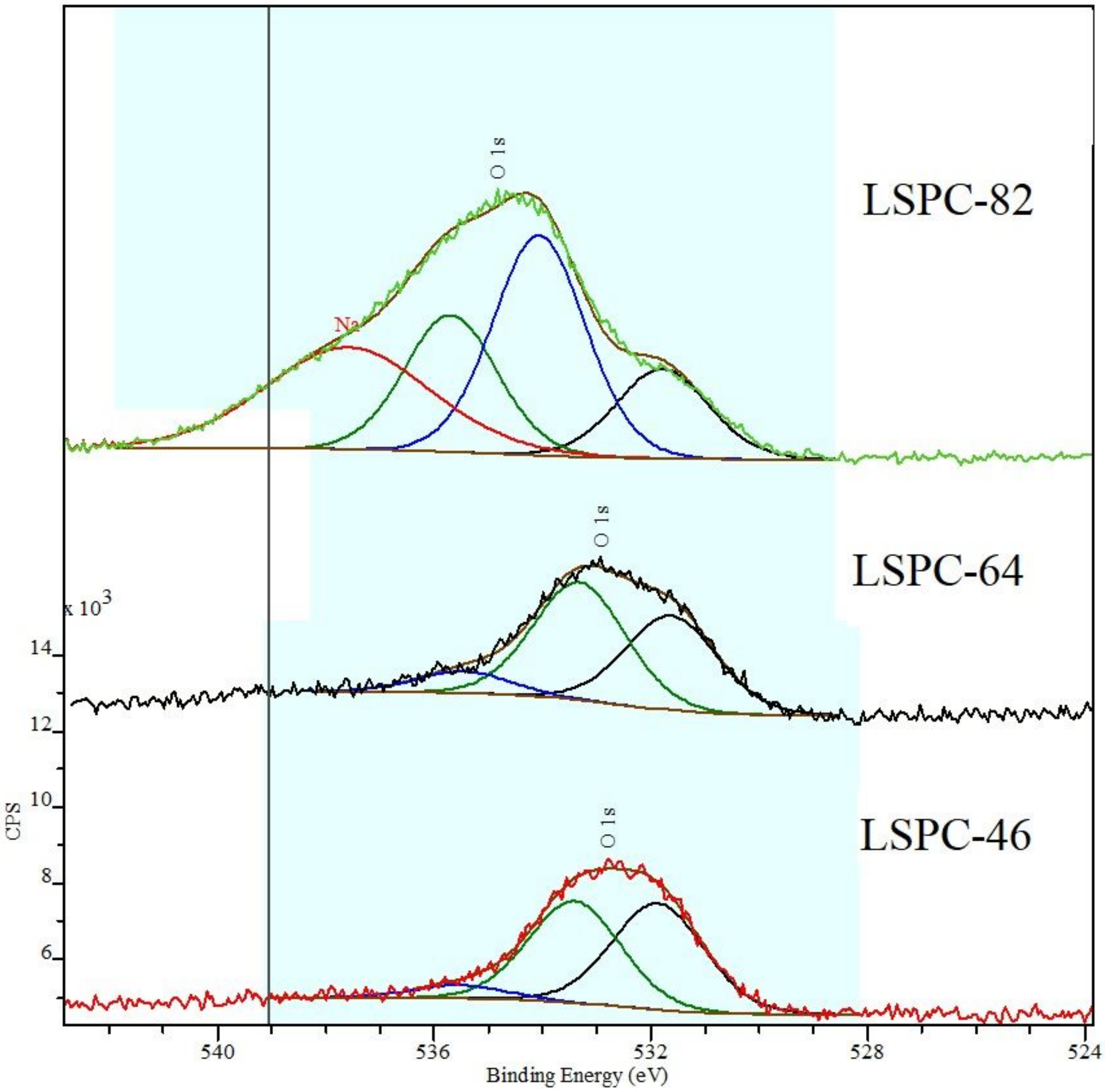

Figure S4: XPS analysis: Detailed O 1s core spectra of LSPCs

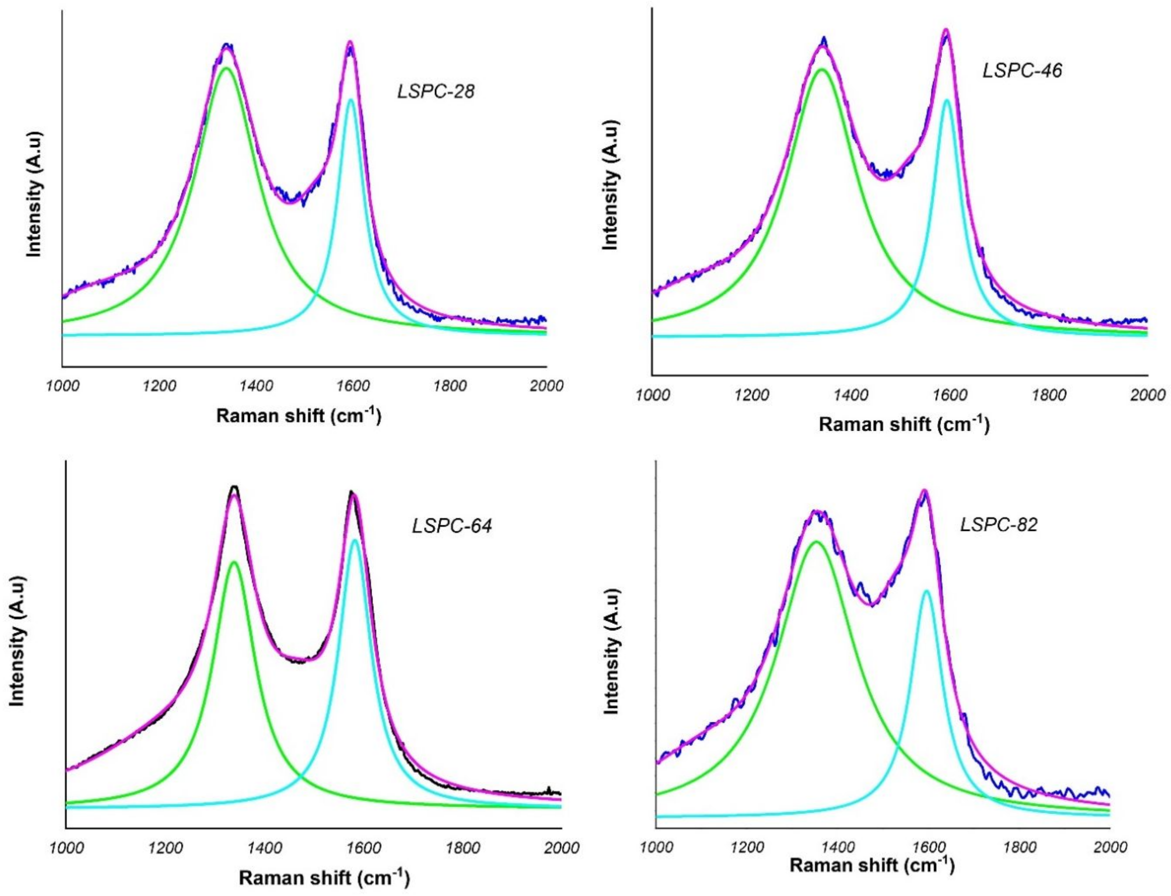

Figure S5: Raman Spectrum of all LSPCs with D and G band fitting

.

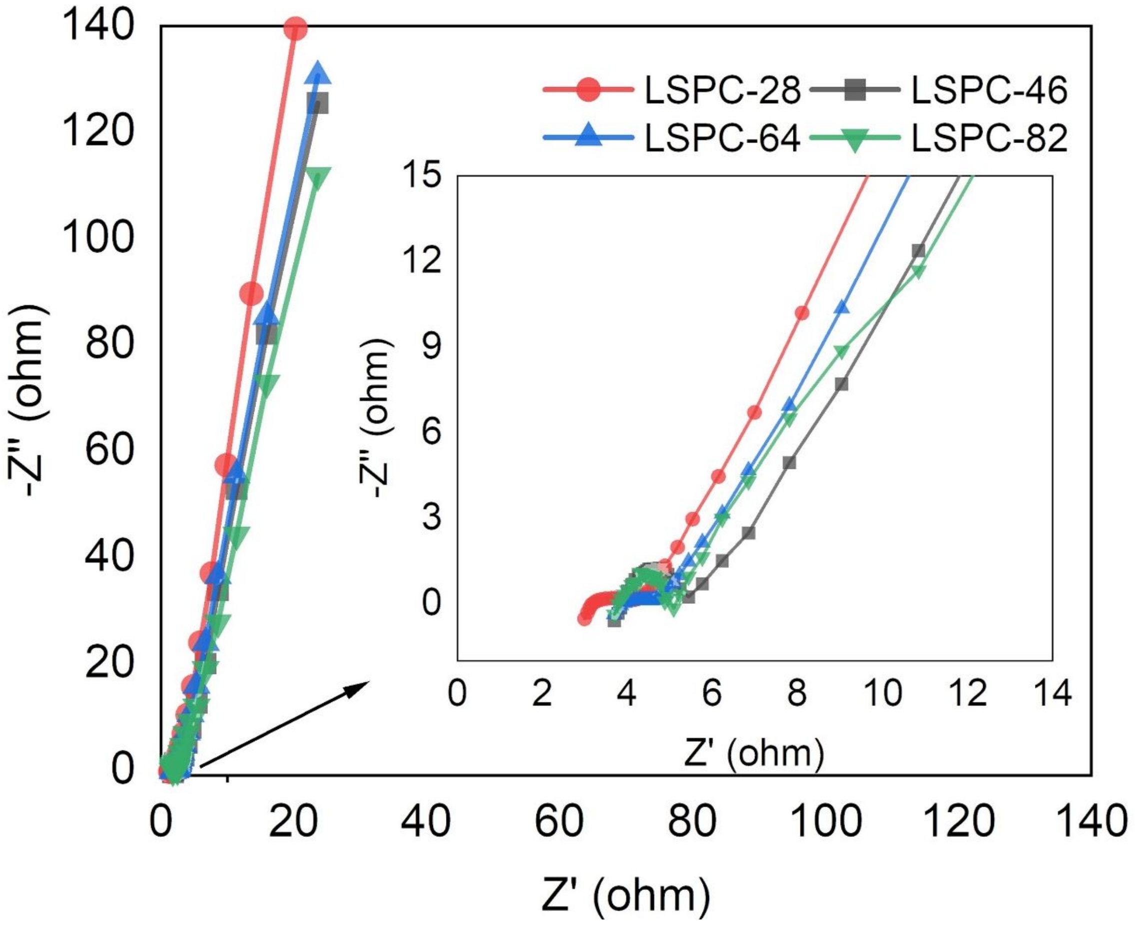

Figure S6: Nyquist plot of LSPCs in electrode configuration 6M KOH electrolyte (10 mHz – 100 KHz)(amplitude 10 mV)

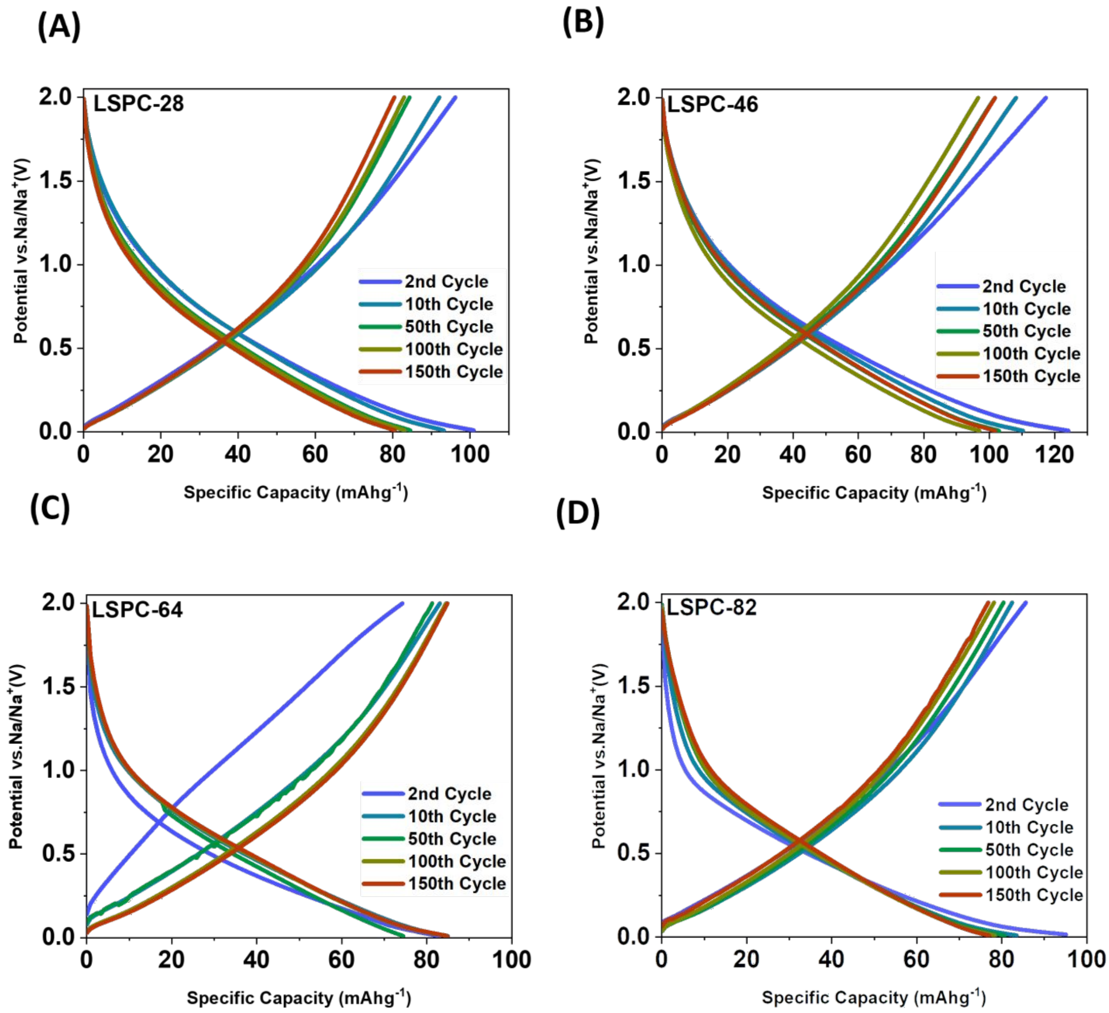

Figure S7: Galvanostatic charge/ discharge curves of LSPCs samples (A) LSPC-28, (B) LSPC-46, (C) LSPC-64, (D) LSPC-82

The yields of carbon samples can vary depending on the specific batch composition, volume of solution, and particularly the PVA content. It's important to note that the sample with higher PVA content yields less carbon, and vice versa. In this study, the sample with low PVA content was giving better performance so yield is not a problem in this technique. For example, for LSPC-28, using a 25 mL solution, we were able to produce spherical beads of ≈9 g that after carbonization yield approximately 2.9 g of LSPC-28. TGA of LSPC-28 results in a ≈32% carbon yield.

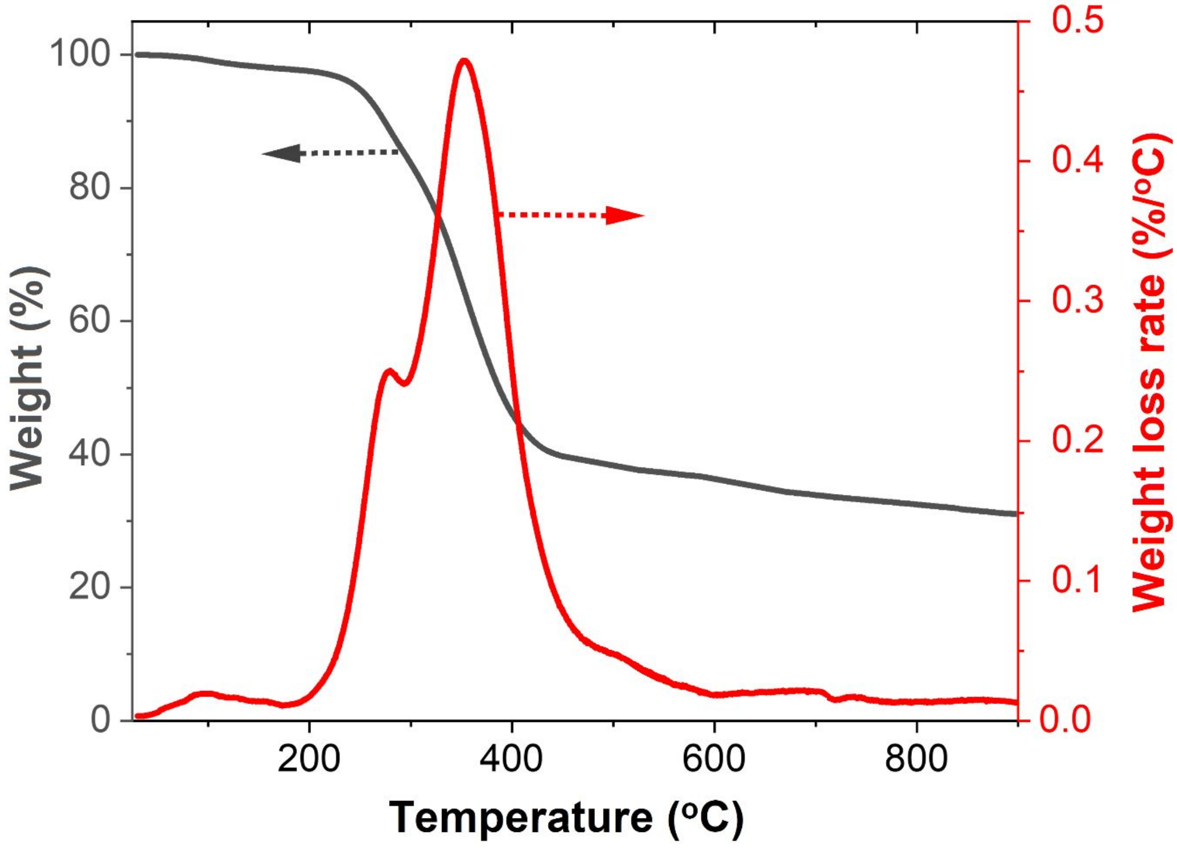

Figure S8: TGA and DTG curve for LSA-28

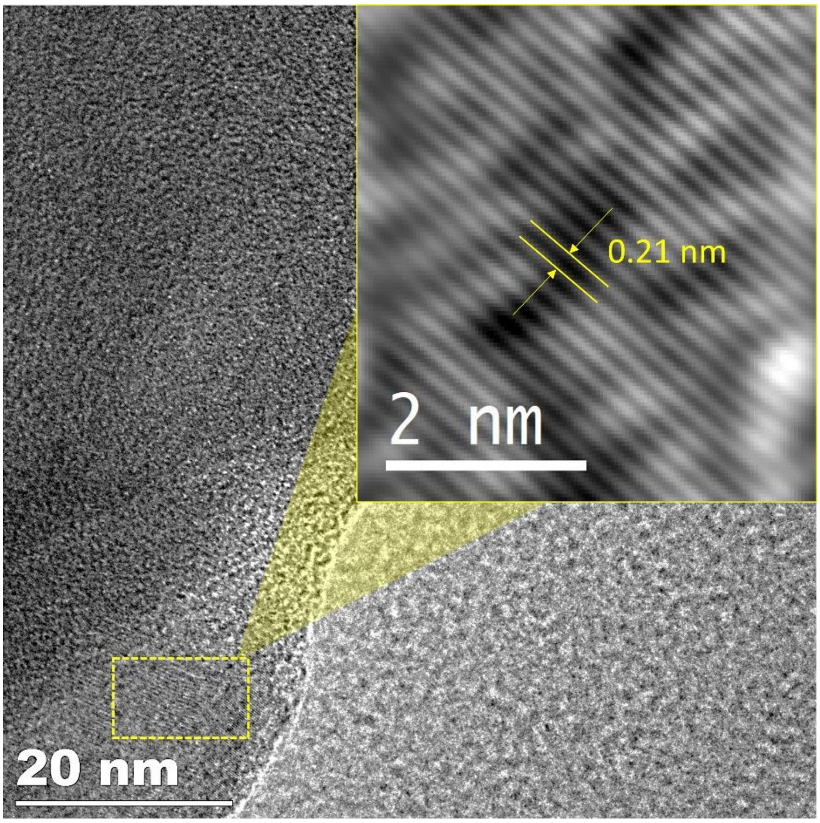

Figure S9: HRTEM image of LSPC-28

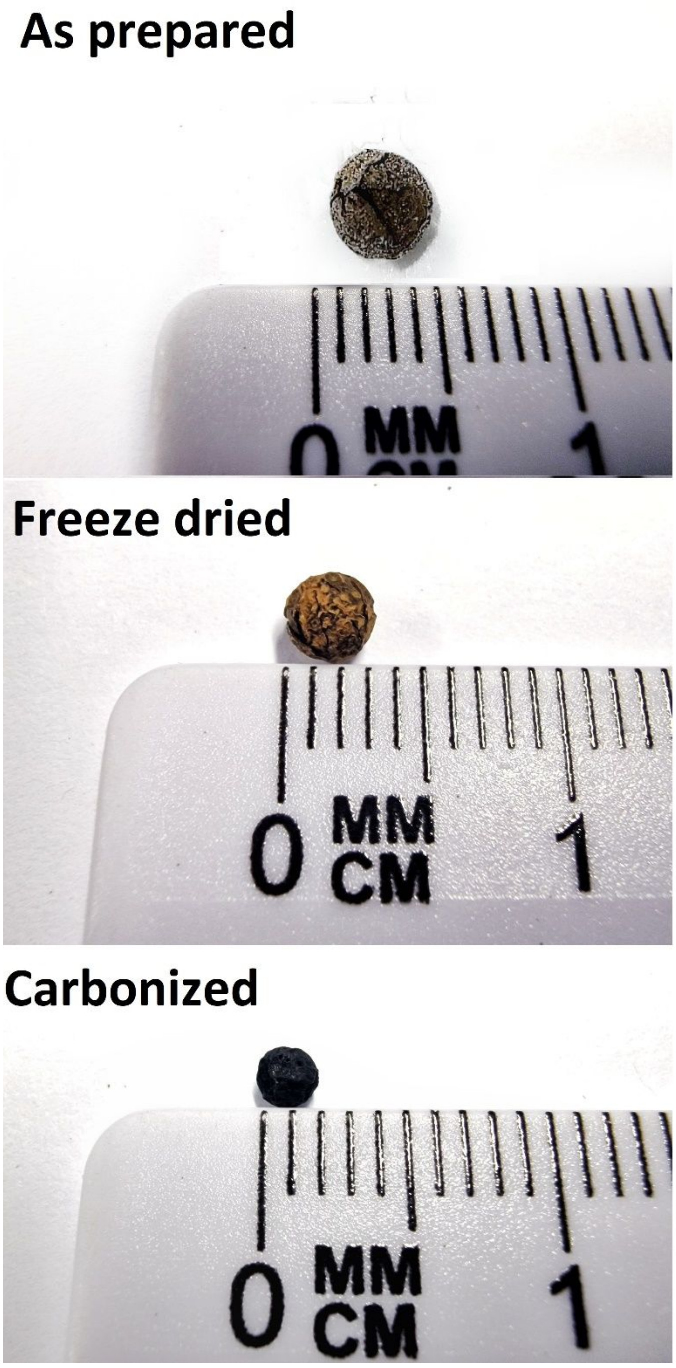

Figure S10: Digital images of LSPC-28 as prepared, freeze-dried, and carbonized.

Table S2: BET Analysis of LSPCs samples

Sample	Surface Area (m^2g^{-1})	Pore diameter (nm)	Pore Volume (cm^3g^{-1})
**LSPC-28**	646	2.095	0.39
**LSPC-46**	426	2.084	0.37
**LSPC-64**	790	2.073	0.68
**LSPC-82**	35.56	-	-

Table S3: Comparison of this study with previous studies

Material and synthesis technique	Supercapacitors			Sodium-ion battery		
	Specific surface area (m^2/g)	Specific capacitance (F/g)	Capacity retention (%)	Specific capacity (mAh/g)	Coulombic efficiency (%)	Ref.
**Alkaline lignin DES treatment, synthetic phenolic resin, twice carbonized**	1026	112.4	97.6 after 10,000 cycles	--	--	[5]
**Acid pretreatment, KOH activation**	1220	118	--	--	--	[6]
**Electrospun carbon nanofiber mats**	583	64	90 after 6000 cycles	--	--	[7]
**Lignin based - reduced graphene oxide, hydrothermal activation**	1804	190	86.5% after 10000 cycles	--	--	[8]
**Lignin based nitrogen-doped carbon composite**	75.3	--	--	193	97.2	[9]
**Lignin based hard carbon material**	6.5	--	--	160	≈99	[10]
**Green synthesis, Lignin spherical porous carbons**	790.6	102.3	106.1 after 5000 cycles	110	98.8	This work

Table S4: Overview of lignin-based anodes in sodium batteries

Sample	Potential window (V)	No of cycles	Reversible Capacity	Rate Capability	Current Density	Capacity Retention	Reference
**Peanut skin**	0.00 to 3.0	200	154 mAh/g	47mAh/g	10A/g	86%	[11]
**Lignin+PAN**	0.00 to 2.5	200	247mAh/g	80mAh/g	1A/g	90.2 %	[12]
**Lignin+PVA**	0.01-2.7	100	248mAh/g	199mAh/g	50mA/g	91%	[13]
**Condensed lignin**	0.005-2.5	500	112mAh/g	116mAh/g	2.5 A/g	46%	[14]
**N-doped porous lignin**	0.01-2.0	1100	100mAh/g	48mAh/g	6.4 A/g	92%	[15]
**Lignin+PVA**	0.01-2.0	150	110mAh/g	130mAh/g	100mA/g	99.9	This work

References:

1. Culebras, M., et al., *Understanding the thermal and dielectric response of organosolv and modified kraft lignin as a carbon fibre precursor.* Green Chemistry, 2018. **20**(19): p. 4461-4472.
2. Beaucamp, A., et al., *Sustainable lignin precursors for tailored porous carbon-based supercapacitor electrodes.* International Journal of Biological Macromolecules, 2022. **221**: p. 1142-1149.

3. Adelnia, H., et al., *Freeze/thawed polyvinyl alcohol hydrogels: Present, past and future.* European Polymer Journal, 2022. **164**: p. 110974.
4. Wu, L., et al., *Synthesis and characterization of biomass lignin-based PVA super-absorbent hydrogel.* International Journal of Biological Macromolecules, 2019. **140**: p. 538-545.
5. Li, P., et al., *Preparation of spherical porous carbon from lignin-derived phenolic resin and its application in supercapacitor electrodes.* International Journal of Biological Macromolecules, 2023. **252**: p. 126271.
6. Martín-Sampedro, R., et al., *Biorefinery of Lignocellulosic Biomass from an Elm Clone: Production of Fermentable Sugars and Lignin-Derived Biochar for Energy and Environmental Applications.* Energy Technology, 2019. **7**(2): p. 277-287.
7. Lai, C., et al., *Free-standing and mechanically flexible mats consisting of electrospun carbon nanofibers made from a natural product of alkali lignin as binder-free electrodes for high-performance supercapacitors.* Journal of Power Sources, 2014. **247**: p. 134-141.
8. Ye, W., et al., *Lignin as a green reductant and morphology directing agent in the fabrication of 3D graphene-based composites for high-performance supercapacitors.* Industrial Crops and Products, 2017. **109**: p. 410-419.
9. Wang, J., et al., *Pyrolysis of Prussian blue for lignin-derived nitrogen-doped biocarbon to boost sodium storage.* Industrial Crops and Products, 2023. **192**: p. 116079.
10. Zhang, H., et al., *Design advanced carbon materials from lignin-based interpenetrating polymer networks for high performance sodium-ion batteries.* Chemical Engineering Journal, 2018. **341**: p. 280-288.
11. Wang, H., et al., *Biomass derived hierarchical porous carbons as high-performance anodes for sodium-ion batteries.* Electrochimica Acta, 2016. **188**: p. 103-110.
12. Jin, J., et al., *Lignin-based electrospun carbon nanofibrous webs as free-standing and binder-free electrodes for sodium ion batteries.* Journal of Power Sources, 2014. **272**: p. 800-807.
13. Zhao, P.-Y., et al., *Electrochemical performance of fulvic acid-based electrospun hard carbon nanofibers as promising anodes for sodium-ion batteries.* Journal of Power Sources, 2016. **334**: p. 170-178.
14. Yoon, D., et al., *Carbon with Expanded and Well-Developed Graphene Planes Derived Directly from Condensed Lignin as a High-Performance Anode for Sodium-Ion Batteries.* ACS Applied Materials & Interfaces, 2018. **10**(1): p. 569-581.
15. Du, L., et al., *Lignin-Derived Nitrogen-Doped Porous Carbon as a High-Rate Anode Material for Sodium Ion Batteries.* Journal of The Electrochemical Society, 2019. **166**(2): p. A423.
